# Molecular Typing of *Mycoplasma genitalium*-Positive Specimens Discriminates between Persistent and Recurrent Infections in Cases of Treatment Failure and Supports Contact Tracing

**DOI:** 10.3390/microorganisms7120609

**Published:** 2019-11-23

**Authors:** Luis Piñeiro, Pedro Idigoras, Gustavo Cilla

**Affiliations:** Microbiology Department, Donostia University Hospital-Biodonostia Health Research Institute, Paseo Dr. Beguiristain s/n, 20014 San Sebastián, Spain

**Keywords:** *Mycoplasma genitalium*, macrolide resistance, persistent/recurrent infections, molecular characterisation, MG191 adhesin, MG309 lipoprotein

## Abstract

*Mycoplasma genitalium* causes a sexually transmitted infection that sometimes persists or recurs despite adequate antibiotic treatment. Between 2014 and 2018, molecular typing was applied to 75 *M. genitalium*-positive samples from 48 patients with repeated infection and/or couples/groups of other infected sexual contacts. MG191 adhesin, MG309 lipoprotein, and the rRNA operon were amplified, sequenced, and typed using phylogenetic, variable number tandem repeat, and single-nucleotide polymorphism analysis, respectively. Amplicons were obtained in 74/75 samples, and the combination of locus patterns gave 44 different genetic profiles (discriminatory index of 0.987), with 43 considering only MG191 and MG309. Interestingly, 15/17 patients who presented a first sample sensitive and a second resistant to macrolides had the same genetic variant in the samples (persistence of the same strain). In 2/17 patients, discordant variants (one mixed infection and one recurrence due to incomplete contact tracing) were detected. In 31 additional not related and randomly distributed samples, MG191 typing obtained 23 different genotypes, with no appreciable clustering over time. The typing method allowed persistent and recurrent infections to be distinguished, indicating that macrolide resistance-associated mutations mostly developed during treatment. To detect these secondary resistant strains, prevent reinfections, and improve the control of *M. genitalium* infections, tests of cure and contact tracing of sexual partners should be mandatory.

## 1. Introduction

*Mycoplasma genitalium* is a leading cause of sexually transmitted infections (STIs), which can sometimes be persistent and/or recurrent due to treatment failure or reinfection, urethritis, cervicitis and pelvic inflammatory disease, among others. The characteristics of this bacterial species (notably, a small genome and no cell wall) make its isolation from culture media difficult and slow, and hence, nucleic acid amplification techniques (NAAT) are used for its detection. Treatment of non-gonococcal urethritis with oral azithromycin 1 g has been associated with variable and growing rates of macrolide resistance in *M. genitalium* [1,2]. Molecular diagnosis of the infection and detection of macrolide resistance-associated mutations with rapid techniques enables targeted therapy [1,3]. Currently, the recommended treatment is oral azithromycin 500 mg on day 1 and 250 mg once daily on days 2 to 5, or moxifloxacin in cases of macrolide resistance [1]. For comprehensive management of this infection, sexual partners should be tested (and as appropriate, treated), and tests of cure (TOC) performed after 3 weeks.

Despite patients adhering to the antibiotic regimen initially prescribed, TOC sometimes reveals that the infection is still present. Four reasons could explain this persistence or recurrence. Persistence may be caused: (1) by relapse with the same strain during treatment due to development of macrolide resistance-associated mutations, or (2) by selection of an initially minority strain in a mixed infection with another sensitive strain. On the other hand, recurrence may be caused: (3) by reinfection with the same strain due to the infected sexual partner not having been treated, or (4) by reinfection with another strain from a new sexual partner. Nonetheless, few studies have assessed the role of these different scenarios in treatment failure. Investigating the molecular epidemiology of *M. genitalium* may shed light on these events and improve control of this infection [4,5].

The objectives of this study were: (a) to assess the discriminatory ability of the various previously described genetic markers and their combinations, for the molecular characterisation of *M. genitalium* from direct sampling; (b) to assess their usefulness for differentiating between persistent and recurrent infections, and to trace the infection through networks of sexual contacts; and (c) to assess the relative importance of the potential causes of treatment failure observed in TOC.

## 2. Materials and Methods

The study was conducted between 2014 and 2018 at Donostia University Hospital (Gipuzkoa, Basque Country, Spain) with a catchment population of 600,000. The samples received for the microbiological diagnosis of patients with suspected STIs were analysed daily using a real-time nucleic acid amplification test (RT-NAAT) that simultaneously detects DNA of *M. genitalium* and six other microorganisms associated with STIs (Allplex^TM^ STI Essential Assay, Seegene). *M. genitalium* DNA-positive samples were analysed again within 24 h with an in-house RT-NAAT (LightCycler, Roche) that uses probes to amplify a fragment of the 23S rRNA gene and differentiate between wild strains and strains with macrolide resistance-associated mutations [3,6]. Patients were given targeted treatment (azithromycin or moxifloxacin) and an appointment for a TOC at 4 to 6 weeks and told to encourage their sexual partners of the last three months to seek testing.

**Figure 1:**
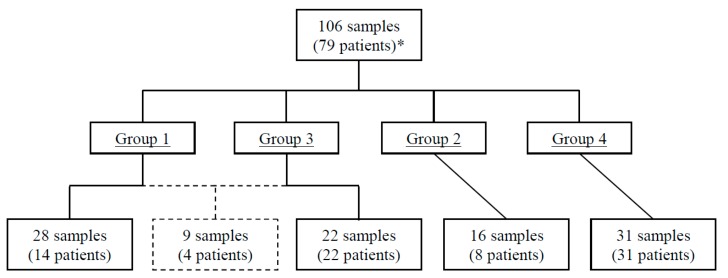
Flow chart of the study population. Group 1: first sample sensitive and second resistant to macrolides. Group 2: first and second samples both sensitive or both resistant. Group 3: 12 couples/groups of 26 sexual contacts (11 cases of two couples and one group of four patients); groups 1 and 3 shared nine samples of four patients (one patient provided 3 samples). Group 4: not related and randomly distributed samples. *65 unrelated patients in the study (34 from groups 1–3 and 31 from group 4).

For the molecular characterisation of *M. genitalium*, we analysed remnant DNA from 106 samples from 79 patients (Figure 1) who were classified into 4 groups: group 1 contained samples from patients with strains initially sensitive to macrolides but later found to be resistant in TOC; group 2 contained samples from patients with strains both sensitive (2a) or both resistant (2b) to macrolides initially and in TOC; group 3 contained samples from patients with couples/groups of sexual contacts; and group 4 contained samples from unrelated patients randomly selected every other month to investigate the genetic diversity and distribution of local strains over time. Samples from groups 1–3 were not selected.

We performed multiple-locus genetic analysis using four variable genetic fragments of *M. genitalium*, the MG191 locus of the *mgpB* adhesin gene (281 bp) [7], the MG309 of the lipoprotein gene (~350 bp) [8] and the internal transcribed spacers (ITS) of the rRNA operon between the 16S and 23S genes (ITS1, 202 bp) and 23 S and 5S genes (ITS2, 228 bp), respectively [8]. The DNA was amplified using polymerase chain reaction (Thermal Cycler, Applied Biosystems) and the amplicons obtained were sequenced bidirectionally (3130XL Genetic Analyzer, Applied Biosystems). The genetic sequences were compared (BLAST, http://www.ncbi.nlm.nih.gov/blast/Blast.cgi) to that of the reference strain of *M. genitalium* G37 (GenBank accession number L43967). Genetic diversity was assessed and different genotypes of each locus identified. For MG191, we performed a phylogenetic study and assigned the numbers previously described for other reference strains to each genotype (genotypes 1–98) [4,9,10,11,12,13,14]. For MG309, we analysed the variable numbers of short tandem repeats and distribution pattern of the trinucleotide repeat units, identifying each strain with this number and pattern. For the ITS1 and ITS2 of the rRNA operon, we assessed the single nucleotide polymorphisms and assigned the previously described numbers to each genotype (1–6 in ITS1 and 1–2 in ITS2) [8]. New consecutive numbering was used for strains found to differ from the known genotypes. Combining these four genotypes, the strains were classified in terms of a genetic profile.

The discriminatory index (DI) of the typing system was calculated by applying Simpson’s diversity index, values >0.90 being considered desirable (Table 1) [15]. The study was approved by the Clinical Research Ethics Committee of Gipuzkoa health region (GMG-2018-01, 09/2018).

## 3. Results

We included 13,135 patients with a suspected STI and found *M. genitalium* DNA in samples from 492 patients (3.75% [95% CI 3.4–4.1%]). There was remnant DNA for assessing macrolide susceptibility in 474 cases. Amplicons were detected in 391 out of 474 cases and macrolide resistance-associated mutations were identified in 68 of them (17.4%). Resistance was detected in the TOC (paired pretreatment macrolide-sensitive strain) in 25 patients (6.4% out of 391 cases overall, 36.8% out of 68 cases of resistance).

For molecular characterisation of the groups, amplification products were obtained in 105 of the 106 samples analysed (Figure 1 and Table S1). In 74 samples from group 1–3 patients, we analysed the four genetic markers and obtained 21 different genotypes (DI of 0.933) in the MG191 locus (Table S2 and Figure S1), 21 (DI of 0.949) in the MG309 locus (Figure S2), three in the ITS1 locus, and two in the ITS2 locus (Table S1). The combination of the genotypes obtained with the four genetic markers (Table 1) yielded 44 different profiles (DI of 0.987): 43 analysing the MG191 and MG309 loci (DI of 0.986), but only four analysing the ITS1 and ITS2 (DI of 0.506).

For analysing group 1, remnant DNA was available from both samples (the initial macrolide-sensitive sample and the TOC sample showing resistance) in 18 out of the 25 cases detected during the study. Amplicons of the four genetic markers were obtained in 36 samples from 17 patients: in one amplicon, amplification was not observed in one of the samples, while in another, it was found in all three samples available. The combination of the four genes identified 20 different profiles in these 36 samples (Table S1). In 15 out of 17 patients (88.2%), the same genetic profile was found in both samples (identical strains in the first test and in the TOC). In the other two patients, the profile differed: in one, the TOC detected a mixed infection (one a sensitive strain and another that was resistant) and in the other, the patient reported a new sexual partner during treatment.

In group 2 (initial and TOC strains both sensitive or both resistant to macrolides), we were able to analyse 8 out of the 12 cases detected and found the same genetic profile in four patients (50%). These patients admitted the possibility of reinfection during treatment before the TOC. In group 3, we found the same profile in 7 out of 12 partners/groups of known sexual contacts (58%). Finally, in group 4 (cases randomly selected), we only analysed MG191 (Figure S1) and found 23 different genotypes in the 31 samples studied.

Analysis of MG191 in all index samples from unrelated patients (*n* = 65) revealed 33 different genotypes (DI of 0.952), with a wide distribution, with only five genotypes being identified in more than two samples and no clustering over time being seen for any genotypes. With this marker, we identified 22 not previously described genotypes, deposited in GenBank (numbered 99–120, Table S1 and Figure S1).

## 4. Discussion

*M. genitalium* infection could be persistent or recurrent due to treatment failure or reinfection [1]. Given the limitations of epidemiological studies, to distinguish between these two scenarios, it would be necessary to undertake molecular analysis. Despite the lack of a standardised scheme for multilocus sequence typing, the typing system used for the molecular characterisation of *M. genitalium* enabled classification of the strains with high discriminatory power. The DIs obtained for MG191 and MG309 were similar to those obtained by Hjorth et al. and Ma et al. [4,9]. Combining the analysis of the MG191 and MG309 loci yielded a DI of 0.986 in the present study, even though some samples/patients were related (groups 1–3). Inclusion of the ITS markers in the typing system only increased the DI slightly (from 0.986 to 0.987).

Our results, from the south of Europe, reflect the fact that *M. genitalium* is genetically diverse, without any pronounced clustering over time, indicating that the infection is endemic in the Basque Country, as described elsewhere [4,8,9,10,11,12,13,14]. The excellent discriminatory power of the method used for charactering the strains and their high genetic diversity allows us to distinguish between persistent and recurrent infections (groups 1 and 2), as well as identify transmission networks (group 3), given the high probability that the strains classified together really are the same.

Despite suitable targeted macrolide treatment in initially sensitive strains, cases of treatment failure have been occasionally observed (~6% in this study) and these may be due to relapse or reinfection (our group 1). Though the emergence of resistance after targeted macrolide treatment has been described previously [3,16,17], possible underlying causes (reinfection or relapse due to induction or selection of resistance during treatment) have rarely been investigated. Our typing method results indicated relapse in 15 out of 17 cases (88.2%), as the initially sensitive strain and the later resistant strain, observed after treatment, had the same genetic profile. This implies that during treatment, the resistance-conferring induction of genetic mutations in the *M. genitalium* 23S rRNA gene is not rare. Jensen et al. and Falk et al. suggested that there was relapse due to resistance induction in seven out of nine and four out of four cases, respectively, using only the MG191 marker [16,17]. Our study considerably increases the number of documented cases of relapse, as strains were characterised using highly discriminatory variable multilocus typing. The strains from the 15 cases of relapse all had different genetic profiles, suggesting that the induced resistance is not clonal.

In other microorganisms, it is known that mutations may appear in the 23S rRNA gene due to macrolide selection pressure and their appearance might depend on the amount of antibiotic exposure [18]. In *M. genitalium*, this type of resistance most commonly develops in patients previously treated with macrolides and given a single oral 1 g dose of azithromycin [1,2,3,19]. Relapses due to selection of a resistant minority variant, initially present in a mixed infection, and reinfection by a different sexual partner with a different strain that was resistant to macrolides, were unusual (1/17 patients for both cases).

Although epidemiological information obtained in TOC is not always completely reliable, in this study, it agreed with the molecular epidemiological data. In group 2, the same genetic profile was identified in the first and second samples in four out of eight patients, and a different profile in the other four. The matching profiles indicate reinfection with the same strain from the same sexual partner who remained untreated (a possibility not ruled out by these patients), while the differing profiles indicate reinfection with another strain from a different partner (>1 sexual partner during treatment being admitted by these patients). Overall, we analysed 25 patients with pre- and post-treatment samples and relapse (*n* = 15) was found to be the main cause of treatment failure at the time of the TOC, this being more common than reinfection (*n* = 9) or the selection of a resistant minority variant (*n* = 1). Finally, in group 3, we detected five couples/groups in which at least one individual had at least one strain that differed from those found in the other(s), indicating incomplete contact tracing of both or all sexual partners.

For cases of suspected STIs, our findings underline the importance not only of rapid NAAT testing to analyse infection by *M. genitalium* and its susceptibility to macrolides to guide targeted treatment, but also of placing emphasis on informing, testing and treating all sexual partners and performing TOC, and in positive cases, retesting macrolide susceptibility to trigger treatment changes if there is resistance (a situation representing approximately one third of the cases of resistance detected in this study, ~6% of all cases). This strategy has been recommended [1,2] given the poor clinical response of *M. genitalium* to doxycycline (30–40%) [20,21,22], although this antibiotic has recently been recommended for the empirical treatment of non-gonococcal urethritis (of unknown aetiology) due to growing resistance to macrolides in *M. genitalium*, attributable to the widespread use of azithromycin 1 g for the treatment of *C. trachomatis* infection [23]. Recently, resistance-guided sequential treatment (doxycycline initially followed by azithromycin or sitafloxacin depending on the resistance test) has shown good efficacy, eradicating the infection and limiting the selection of antibiotic resistance [24]. This strategy could be a possibility in the context of high macrolide resistance rates and/or when guided therapy cannot be applied rapidly. However, limited adherence to a multiple-dose antibiotic regimen [25,26], as well as possible adverse events and/or selection of new antibiotic resistances due to unnecessary overtreatment with doxycycline in macrolide-susceptible infections, should be considered.

Among the limitations of this study, we should highlight the following. (A) The NAAT used for detecting resistance may have limited sensitivity, especially for samples with a low bacterial load [3,6]; consequently, it might not detect minority resistant bacterial populations in pre-treatment samples, and hence, what appeared to be induction would actually be selection of resistance, although in both cases it would be relapse. (B) Naturally-occurring mutations have been previously described in the lipoprotein gene [8,9], attributable to slipped strand mispairing during DNA replication [27]; but their occurrence would have led to similar strains being classified as different, and hence, this does not alter the classification of relapse. (C) Around 60% of patients returned for TOC, meaning that the resistance induction rate may be underestimated, although we assume that the infection had clinically resolved in most of the patients who did not attend these tests. And (D) some interpretations of the findings of the study are based on a limited number of samples.

## 5. Conclusions

The typing system used allowed us to widen the knowledge of the molecular epidemiology of *M. genitalium* infection and document the dominant role of relapse rather than reinfection in treatment failure, which is attributable to resistance induction during macrolide treatment. These findings underline that to improve the control of this infection there is a need to adhere to a combined management strategy that includes the detection of *M. genitalium* and analysis of macrolide susceptibility with rapid NAAT, use of targeted antibiotics, testing and treatment of sexual partners, and TOC.

## Figures and Tables

**Table 1:** Discriminatory indices obtained in the study groups (G1–3, *n* = 74 and G4, *n* = 31) using different genes of *Mycoplasma genitalium* or their combinations.

Study Group(s) (Method)	No. of Types	Size (%) of Largest Type	Discriminatory Index
G1–3 (Adh+Lip+ITS1+ITS2)	44	4	0.9871
G1–3 (Adh+Lip)	43	4	0.9863
G1–3 (ITS1+ITS2)	4	64	0.5056
G1–3 (Adh)	21	14	0.9334
G1–3 (Lip)	21	9	0.9496
G4 (Adh)	23	16	0.9634
G1–4 (Adh) *	33	14	0.9524

G1: first sample sensitive and second resistant to macrolides. G2: first and second samples both sensitive or both resistant. G3: couples/groups of sexual contacts. G4: not related and randomly distributed samples. Adh: adhesin MG191 *mgpB* gene. Lip: lipoprotein MG309 gene. ITS: internal transcribed spacers of rRNA operon. * Index samples from unrelated patients (*n* = 65).

## Supplementary Materials

The following are available online at https://www.mdpi.com/2076-2607/7/12/609/s1, Table S1: Main epidemiological and molecular typing data. Table S2: Fasta sequences and MultAlin analysis of the *mgpB* adhesin gene. Figure S1: Phylogenetic analysis of the adhesin *mgpB* gene. Figure S2: Variable number tandem repeat analysis of lipoprotein gene.
