# Supplementary material for: Molecular Typing of Mycoplasma genitalium-Positive Specimens Discriminates between Persistent and Recurrent Infections in Cases of Treatment Failure and Supports Contact Tracing
Source: Microorganisms. 2019 Nov 23;7(12):609. doi: 10.3390/microorganisms7120609 (PMC6955785; doi:10.3390/microorganisms7120609)
Supplement: Supplementary file 1 [file microorganisms-07-00609-s001.zip › microorganisms-621513-figures.zip!/1.Fig1.pdf]

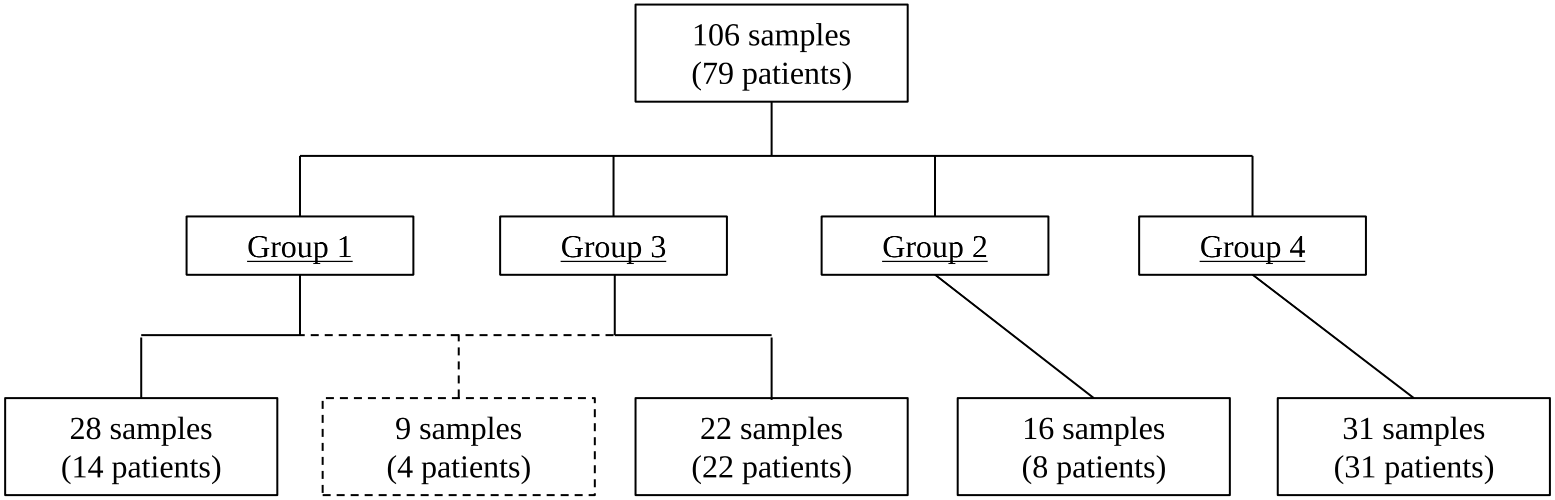
106 samples
(79 patients)
Group 1
Group 3
Group 2
Group 4
28 samples
(14 patients)
9 samples
(4 patients)
22 samples
(22 patients)
16 samples
(8 patients)
31 samples
(31 patients)
