# Supplementary material for: Molecular Typing of Mycoplasma genitalium-Positive Specimens Discriminates between Persistent and Recurrent Infections in Cases of Treatment Failure and Supports Contact Tracing
Source: Microorganisms. 2019 Nov 23;7(12):609. doi: 10.3390/microorganisms7120609 (PMC6955785; doi:10.3390/microorganisms7120609)
Supplement: Supplementary file 1 [file microorganisms-07-00609-s001.zip › microorganisms-621513-figures.zip!/2.FigS1.pdf]

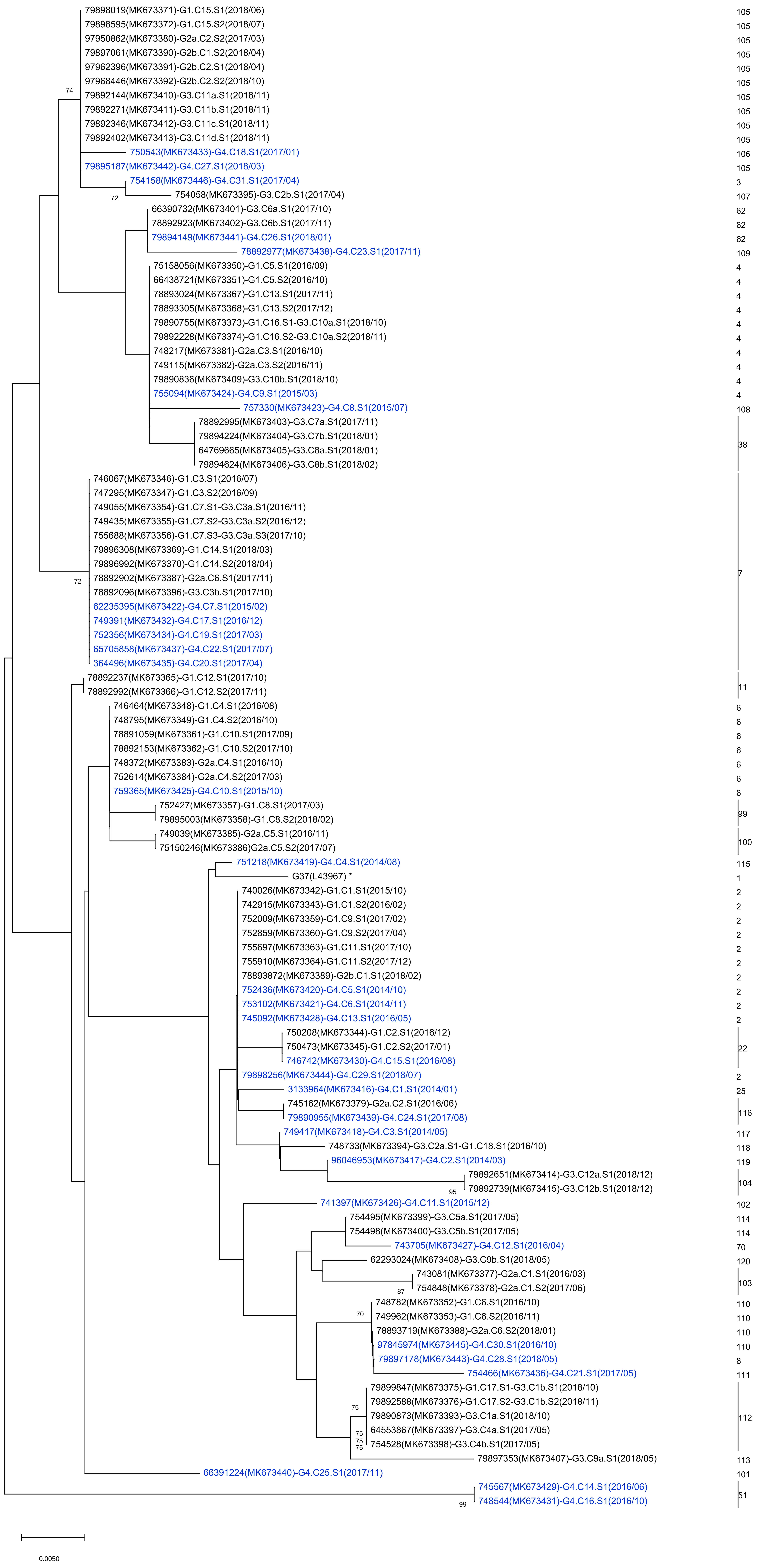

79898019(MK673371)-G1.C15.S1(2018/06) 105
79898595(MK673372)-G1.C15.S2(2018/07) 105
97950862(MK673380)-G2a.C2.S2(2017/03) 105
79897061(MK673390)-G2b.C1.S2(2018/04) 105
97962396(MK673391)-G2b.C2.S1(2018/04) 105
97968446(MK673392)-G2b.C2.S2(2018/10) 105
74
79892144(MK673410)-G3.C11a.S1(2018/11) 105
79892271(MK673411)-G3.C11b.S1(2018/11) 105
79892346(MK673412)-G3.C11c.S1(2018/11) 105
79892402(MK673413)-G3.C11d.S1(2018/11) 105
750543(MK673433)-G4.C18.S1(2017/01) 106
79895187(MK673442)-G4.C27.S1(2018/03) 105
754158(MK673446)-G4.C31.S1(2017/04) 3
72
754058(MK673395)-G3.C2b.S1(2017/04) 107
66390732(MK673401)-G3.C6a.S1(2017/10) 62
78892923(MK673402)-G3.C6b.S1(2017/11) 62
79894149(MK673441)-G4.C26.S1(2018/01) 62
78892977(MK673438)-G4.C23.S1(2017/11) 109
75158056(MK673350)-G1.C5.S1(2016/09) 4
66438721(MK673351)-G1.C5.S2(2016/10) 4
78893024(MK673367)-G1.C13.S1(2017/11) 4
78893305(MK673368)-G1.C13.S2(2017/12) 4
79890755(MK673373)-G1.C16.S1-G3.C10a.S1(2018/10) 4
79892228(MK673374)-G1.C16.S2-G3.C10a.S2(2018/11) 4
748217(MK673381)-G2a.C3.S1(2016/10) 4
749115(MK673382)-G2a.C3.S2(2016/11) 4
79890836(MK673409)-G3.C10b.S1(2018/10) 4
755094(MK673424)-G4.C9.S1(2015/03) 4
757330(MK673423)-G4.C8.S1(2015/07) 108
78892995(MK673403)-G3.C7a.S1(2017/11)
79894224(MK673404)-G3.C7b.S1(2018/01)
64769665(MK673405)-G3.C8a.S1(2018/01)
79894624(MK673406)-G3.C8b.S1(2018/02)
38
746067(MK673346)-G1.C3.S1(2016/07)
747295(MK673347)-G1.C3.S2(2016/09)
749055(MK673354)-G1.C7.S1-G3.C3a.S1(2016/11)
749435(MK673355)-G1.C7.S2-G3.C3a.S2(2016/12)
755688(MK673356)-G1.C7.S3-G3.C3a.S3(2017/10)
79896308(MK673369)-G1.C14.S1(2018/03)
79896992(MK673370)-G1.C14.S2(2018/04)
72
78892902(MK673387)-G2a.C6.S1(2017/11)
78892096(MK673396)-G3.C3b.S1(2017/10)
62235395(MK673422)-G4.C7.S1(2015/02)
749391(MK673432)-G4.C17.S1(2016/12)
752356(MK673434)-G4.C19.S1(2017/03)
65705858(MK673437)-G4.C22.S1(2017/07)
364496(MK673435)-G4.C20.S1(2017/04)
7
78892237(MK673365)-G1.C12.S1(2017/10)
78892992(MK673366)-G1.C12.S2(2017/11)
11
746464(MK673348)-G1.C4.S1(2016/08) 6
748795(MK673349)-G1.C4.S2(2016/10) 6
78891059(MK673361)-G1.C10.S1(2017/09) 6
78892153(MK673362)-G1.C10.S2(2017/10) 6
748372(MK673383)-G2a.C4.S1(2016/10) 6
752614(MK673384)-G2a.C4.S2(2017/03) 6
759365(MK673425)-G4.C10.S1(2015/10) 6
752427(MK673357)-G1.C8.S1(2017/03)
79895003(MK673358)-G1.C8.S2(2018/02)
99
749039(MK673385)-G2a.C5.S1(2016/11)
75150246(MK673386)G2a.C5.S2(2017/07)
100
751218(MK673419)-G4.C4.S1(2014/08) 115
G37(L43967) * 1
740026(MK673342)-G1.C1.S1(2015/10) 2
742915(MK673343)-G1.C1.S2(2016/02) 2
752009(MK673359)-G1.C9.S1(2017/02) 2
752859(MK673360)-G1.C9.S2(2017/04) 2
755697(MK673363)-G1.C11.S1(2017/10) 2
755910(MK673364)-G1.C11.S2(2017/12) 2
78893872(MK673389)-G2b.C1.S1(2018/02) 2
752436(MK673420)-G4.C5.S1(2014/10) 2
753102(MK673421)-G4.C6.S1(2014/11) 2
745092(MK673428)-G4.C13.S1(2016/05) 2
750208(MK673344)-G1.C2.S1(2016/12)
750473(MK673345)-G1.C2.S2(2017/01)
746742(MK673430)-G4.C15.S1(2016/08)
22
79898256(MK673444)-G4.C29.S1(2018/07) 2
3133964(MK673416)-G4.C1.S1(2014/01) 25
745162(MK673379)-G2a.C2.S1(2016/06)
79890955(MK673439)-G4.C24.S1(2017/08)
116
749417(MK673418)-G4.C3.S1(2014/05) 117
748733(MK673394)-G3.C2a.S1-G1.C18.S1(2016/10) 118
96046953(MK673417)-G4.C2.S1(2014/03) 119
79892651(MK673414)-G3.C12a.S1(2018/12)
95
79892739(MK673415)-G3.C12b.S1(2018/12)
104
741397(MK673426)-G4.C11.S1(2015/12) 102
754495(MK673399)-G3.C5a.S1(2017/05) 114
754498(MK673400)-G3.C5b.S1(2017/05) 114
743705(MK673427)-G4.C12.S1(2016/04) 70
62293024(MK673408)-G3.C9b.S1(2018/05) 120
743081(MK673377)-G2a.C1.S1(2016/03)
87
754848(MK673378)-G2a.C1.S2(2017/06)
103
70
748782(MK673352)-G1.C6.S1(2016/10) 110
749962(MK673353)-G1.C6.S2(2016/11) 110
78893719(MK673388)-G2a.C6.S2(2018/01) 110
97845974(MK673445)-G4.C30.S1(2016/10) 110
79897178(MK673443)-G4.C28.S1(2018/05) 8
754466(MK673436)-G4.C21.S1(2017/05) 111
79899847(MK673375)-G1.C17.S1-G3.C1b.S1(2018/10)
79892588(MK673376)-G1.C17.S2-G3.C1b.S2(2018/11)
75
79890873(MK673393)-G3.C1a.S1(2018/10)
64553867(MK673397)-G3.C4a.S1(2017/05)
75
75
75
754528(MK673398)-G3.C4b.S1(2017/05)
112
79897353(MK673407)-G3.C9a.S1(2018/05) 113
66391224(MK673440)-G4.C25.S1(2017/11) 101
745567(MK673429)-G4.C14.S1(2016/06)
99
748544(MK673431)-G4.C16.S1(2016/10)
51
0.0050
