# Supplementary material for: Molecular Typing of Mycoplasma genitalium-Positive Specimens Discriminates between Persistent and Recurrent Infections in Cases of Treatment Failure and Supports Contact Tracing
Source: Microorganisms. 2019 Nov 23;7(12):609. doi: 10.3390/microorganisms7120609 (PMC6955785; doi:10.3390/microorganisms7120609)
Supplement: Supplementary file 1 [file microorganisms-07-00609-s001.zip › microorganisms-621513-figures.zip!/3.FigS2.pdf]

No. (%) of samples	No. of repeats	AAAAGGATC	ACT	ACT	ACT	ACT	ATT	x x x	x x x	x x x	x x x	x x x	ACT	ATT	ACT	ATT	ACT	ATT	ACT	TGTATTTGA *
5 (6,8)	9	AAAAGGATC	ACT	ACT	x x x	x x x	x x x	x x x	x x x	x x x	x x x	x x x	ACT	ACT	ACT	ATT	ACT	ATT	ACT	TGTATTTGA
7 (9,5)	10a	AAAAGGATC	ACT	ACT	ACT	x x x	x x x	x x x	x x x	x x x	x x x	x x x	ACT	ACT	ACT	ATT	ACT	ATT	ACT	TGTATTTGA
1 (1,4)	10b	AAAAGGATC	ACT	ACT	ACT	x x x	x x x	x x x	x x x	x x x	x x x	x x x	ACT	ACT	ATT	ATT	ACT	ATT	ACT	TGTATTTGA
7 (9,5)	11a	AAAAGGATC	ACT	ACT	ACT	ACT	x x x	x x x	x x x	x x x	x x x	x x x	ACT	ACT	ACT	ATT	ACT	ATT	ACT	TGTATTTGA
5 (6,8)	11b	AAAAGGATC	ACT	ACT	ACT	ACT	x x x	x x x	x x x	x x x	x x x	x x x	ACT	ATT	ACT	ATT	ACT	ATT	ACT	TGTATTTGA
1 (1,4)	11c	AAAAGGATC	ACT	ACT	ACT	ACT	x x x	x x x	x x x	x x x	x x x	x x x	ACT	ACT	ACT	ACT	ACT	ATT	ACT	TGTATTTGA
5 (6,8)	12a	AAAAGGATC	ACT	ACT	ACT	ACT	ACT	x x x	x x x	x x x	x x x	x x x	ACT	ACT	ACT	ATT	ACT	ATT	ACT	TGTATTTGA
2 (2,7)	12b	AAAAGGATC	ACT	ACT	ATT	ACT	ATT	x x x	x x x	x x x	x x x	x x x	ACT	ATT	ACT	ATT	ACT	ATT	ACT	TGTATTTGA
1 (1,4)	12c	AAAAGGATC	ACT	ACT	ACT	ACT	ACT	x x x	x x x	x x x	x x x	x x x	ACT	ATT	ACT	ATT	ACT	ATT	ACT	TGTATTTGA
3 (4,1)	13a	AAAAGGATC	ACT	ACT	ACT	ACT	ACT	ACT	x x x	x x x	x x x	x x x	ACT	ACT	ACT	ATT	ACT	ATT	ACT	TGTATTTGA
2 (2,7)	13b	AAAAGGATC	ACT	ACT	ACT	ACT	ACT	ACT	x x x	x x x	x x x	x x x	ACT	ATT	ACT	ATT	ACT	ATT	ACT	TGTATTTGA
3 (4,1)	14a	AAAAGGATC	ACT	ACT	ACT	ACT	ACT	ACT	ACT	x x x	x x x	x x x	ACT	ACT	ACT	ATT	ACT	ATT	ACT	TGTATTTGA
7 (9,5)	14b	AAAAGGATC	ACT	ACT	ACT	ACT	ACT	ACT	ATT	x x x	x x x	x x x	ACT	ATT	ACT	ATT	ACT	ATT	ACT	TGTATTTGA
2 (2,7)	14c	AAAAGGATC	ACT	ACT	ACT	ATT	ACT	ACT	ACT	x x x	x x x	x x x	ACT	ACT	ACT	ATT	ACT	ATT	ACT	TGTATTTGA
1 (1,4)	14d	AAAAGGATC	ACT	ACT	ACT	ACT	ACT	ACT	ACT	x x x	x x x	x x x	ACT	ATT	ACT	ATT	ACT	ATT	ACT	TGTATTTGA
4 (5,4)	15a	AAAAGGATC	ACT	ACT	ACT	ACT	ACT	ACT	ACT	ACT	x x x	x x x	ACT	ATT	ACT	ATT	ACT	ATT	ACT	TGTATTTGA
3 (4,1)	15b	AAAAGGATC	ACT	ACT	ACT	ACT	ACT	ACT	ACT	ATT	x x x	x x x	ACT	ATT	ACT	ATT	ACT	ATT	ACT	TGTATTTGA
6 (8,1)	16a	AAAAGGATC	ACT	ACT	ACT	ACT	ACT	ACT	ACT	ACT	ACT	x x x	ACT	ACT	ACT	ATT	ACT	ATT	ACT	TGTATTTGA
2 (2,7)	16b	AAAAGGATC	ACT	ACT	ACT	ACT	ACT	ACT	ACT	ACT	ACT	x x x	ACT	ATT	ACT	ATT	ACT	ATT	ACT	TGTATTTGA
4 (5,4)	17a	AAAAGGATC	ACT	ACT	ACT	ACT	ACT	ACT	ACT	ACT	ACT	ATT	ACT	ATT	ACT	ATT	ACT	ATT	ACT	TGTATTTGA
3 (4,1)	17b	AAAAGGATC	ACT	ACT	ACT	ACT	ACT	ACT	ACT	ACT	ACT	ACT	ACT	ATT	ACT	ATT	ACT	ATT	ACT	TGTATTTGA
