# Supplementary material for: Molecular Typing of Mycoplasma genitalium-Positive Specimens Discriminates between Persistent and Recurrent Infections in Cases of Treatment Failure and Supports Contact Tracing
Source: Microorganisms. 2019 Nov 23;7(12):609. doi: 10.3390/microorganisms7120609 (PMC6955785; doi:10.3390/microorganisms7120609)
Supplement: Supplementary file 1 [file microorganisms-07-00609-s001.zip › microorganisms-621513-supple.zip!/1.TabS1.docx]

**Table S1.** Main epidemiological data on 79 patients with infection by *Mycoplasma genitalium* (Gipuzkoa, Spain 2014-2018), classified by study group (G), case (C) and sample (S) number in each group; and molecular data on the 106 strains analysed.

|  |  |  |  | **Genetic characterisation** | | | | | | | | |
| --- | --- | --- | --- | --- | --- | --- | --- | --- | --- | --- | --- | --- |
| **Sex** | **Age** | **Sample** | **Date** | **Study group** | **Sample no.** | **Adhesin GenBank no.** | **MG191 type** | **MG309 type** | **ITS1 type** | **ITS2 type** | **Marker pattern** | **Genetic profile** |
| 1 | 20 | Urethra | 30 Oct. 2015 | G1-C1.S1 | 740026 | MK673342 | 2 | 9 | 2 | 1 | 2-09-2-1 | 1 |
| 1 | 20 | Urethra | 11 Feb. 2016 | G1-C1.S2 | 742915 | MK673343 | 2 | 9 | 2 | 1 | 2-09-2-1 | 1 |
| 1 | 33 | Urethra | 16 Dec. 2016 | G1-C2.S1 | 750208 | MK673344 | 22 | 14b | 2 | 1 | 22-14b-2-1 | 2 |
| 1 | 33 | Urethra | 12 Jan. 2017 | G1-C2.S2 | 750473 | MK673345 | 22 | 14b | 2 | 1 | 22-14b-2-1 | 2 |
| 1 | 39 | Urethra | 7 Jul. 2016 | G1-C3.S1 | 746067 | MK673346 | 7 | 14a | 3 | 1 | 7-14a-3-1 | 3 |
| 1 | 39 | Urethra | 1 Sep. 2016 | G1-C3.S2 | 747295 | MK673347 | 7 | 14a | 3 | 1 | 7-14a-3-1 | 3 |
| 1 | 35 | Urethra | 10 Aug. 2016 | G1-C4.S1 | 746464 | MK673348 | 6 | 17a | 2 | 1 | 6-17a-2-1 | 4 |
| 1 | 35 | Urethra | 27 Oct. 2016 | G1-C4.S2 | 748795 | MK673349 | 6 | 17a | 2 | 1 | 6-17a-2-1 | 4 |
| 2 | 24 | Cervix | 6 Sep. 2016 | G1-C5.S1 | 75158056 | MK673350 | 4 | 11a | 2 | 1 | 4-11a-2-1 | 5 |
| 2 | 24 | Cervix | 18 Oct. 2016 | G1-C5.S2 | 66438721 | MK673351 | 4 | 13a | 2 | 1 | 4-13a-2-1 | 6 |
| 1 | 22 | Urethra | 26 Oct. 2016 | G1-C6.S1 | 748782 | MK673352 | 110 | 10a | 2 | 1 | 110-10a-2-1 | 7 |
| 1 | 22 | Urethra | 28 Nov. 2016 | G1-C6.S2 | 749962 | MK673353 | 110 | 10a | 2 | 1 | 110-10a-2-1 | 7 |
| 2 | 36 | Cervix | 8 Nov. 2016 | G1-C7.S1, G3-C3a.S1 | 749055 | MK673354 | 7 | 16a | 3 | 1 | 7-16a-3-1 | 8 |
| 2 | 36 | Cervix | 7 Dec. 2016 | G1-C7.S2, G3-C3a.S2 | 749435 | MK673355 | 7 | 16a | 3 | 1 | 7-16a-3-1 | 8 |
| 2 | 37 | Cervix | 3 Oct. 2017 | G1-C7.S3, G3-C3a.S3 | 755688 | MK673356 | 7 | 16a | 3 | 1 | 7-16a-3-1 | 8 |
| 1 | 36 | Urethra | 9 Mar. 2017 | G1-C8.S1 | 752427 | MK673357 | 99 | 15b | 2 | 1 | 99-15b-2-1 | 9 |
| 1 | 37 | Urine | 21 Feb. 2018 | G1-C8.S2 | 79895003 | MK673358 | 99 | 15b | 2 | 1 | 99-15b-2-1 | 9 |
| 1 | 35 | Urethra | 14 Feb. 2017 | G1-C9.S1 | 752009 | MK673359 | 2 | 15a | 2 | 1 | 2-15a-2-1 | 10 |
| 1 | 35 | Urethra | 3 Apr. 2017 | G1-C9.S2 | 752859 | MK673360 | 2 | 16b | 2 | 1 | 2-16b-2-1 | 11 |
| 1 | 30 | Urethra | 1 Sep. 2017 | G1-C10.S1 | 78891059 | MK673361 | 6 | 14b | 2 | 1 | 6-14b-2-1 | 12 |
| 1 | 30 | Urethra | 9 Oct. 2017 | G1-C10.S2 | 78892153 | MK673362 | 6 | 14b | 2 | 1 | 6-14b-2-1 | 12 |
| 2 | 27 | Vagina | 4 Oct. 2017 | G1-C11.S1 | 755697 | MK673363 | 2 | 13b | 2 | 2 | 2-13b-2-2 | 13 |
| 2 | 27 | Cervix | 4 Dec. 2017 | G1-C11.S2 | 755910 | MK673364 | 2 | 13b | 2 | 2 | 2-13b-2-2 | 13 |
| 1 | 24 | Urethra | 13 Oct. 2017 | G1-C12.S1 | 78892237 | MK673365 | 11 | 11b | 3 | 1 | 11-11b-3-1 | 14 |
| 1 | 24 | Urethra | 20 Nov. 2017 | G1-C12.S2 | 78892992 | MK673366 | 11 | 11b | 3 | 1 | 11-11b-3-1 | 14 |
| 1 | 25 | Urethra | 28 Nov. 2017 | G1-C13.S1 | 78893024 | MK673367 | 4 | 14c | 2 | 1 | 4-14c-2-1 | 15 |
| 1 | 25 | Urethra | 14 Dec. 2017 | G1-C13.S2 | 78893305 | MK673368 | 4 | 14c | 2 | 1 | 4-14c-2-1 | 15 |
| 2 | 38 | Vagina | 16 Mar. 2018 | G1-C14.S1 | 79896308 | MK673369 | 7 | 11a | 3 | 1 | 7-11a-3-1 | 16 |
| 2 | 38 | Vagina | 25 Apr. 2018 | G1-C14.S2 | 79896992 | MK673370 | 7 | 11a | 3 | 1 | 7-11a-3-1 | 16 |
| 1 | 25 | Urine | 19 Jun. 2018 | G1-C15.S1 | 79898019 | MK673371 | 105 | 12a | 3 | 1 | 105-12a-3-1 | 17 |
| 1 | 25 | Urethra | 27 Jul. 2018 | G1-C15.S2 | 79898595 | MK673372 | 105 | 12a | 3 | 1 | 105-12a-3-1 | 17 |
| 1 | 25 | Urethra | 4 Oct. 2018 | G1-C16.S1, G3-C10a.S1 | 79890755 | MK673373 | 4 | 11b | 2 | 1 | 4-11b-2-1 | 18 |
| 1 | 25 | Urethra | 12 Nov. 2018 | G1-C16.S2, G3-C10a.S2 | 79892228 | MK673374 | 4 | 11b | 2 | 1 | 4-11b-2-1 | 18 |
| 2 | 35 | Vagina | 25 Oct. 2018 | G1-C17.S1, G3-C1b.S1 | 79899847 | MK673375 | 112 | 17b | 2 | 1 | 112-17b-2-1 | 19 |
| 2 | 36 | Vagina | 29 Nov. 2018 | G1-C17.S2, G3-C1b.S2 | 79892588 | MK673376 | 112 | 17b | 2 | 1 | 112-17b-2-1 | 19 |
| 1 | 67 | Urethra | 10 Mar. 2016 | G2a-C1.S1 | 743081 | MK673377 | 103 | 10a | 2 | 1 | 103-10a-2-1 | 20 |
| 1 | 68 | Urethra | 1 Jun. 2017 | G2a-C1.S2 | 754848 | MK673378 | 103 | 10a | 2 | 1 | 103-10a-2-1 | 20 |
| 1 | 26 | Urethra | 1 Jun. 2016 | G2a-C2.S1 | 745162 | MK673379 | 116 | 9 | 3 | 1 | 116-09-3-1 | 21 |
| 1 | 27 | Urethra | 8 Mar. 2017 | G2a-C2.S2 | 97950862 | MK673380 | 105 | 11c | 3 | 1 | 105-11c-3-1 | 22 |
| 2 | 21 | Cervix | 14 Oct. 2016 | G2a-C3.S1 | 748217 | MK673381 | 4 | 12a | 3 | 1 | 4-12a-3-1 | 23 |
| 2 | 21 | Cervix | 12 Nov. 2016 | G2a-C3.S2 | 749115 | MK673382 | 4 | 12a | 3 | 1 | 4-12a-3-1 | 23 |
| 2 | 24 | Cervix | 26 Oct. 2016 | G2a-C4.S1 | 748372 | MK673383 | 6 | 12b | 2 | 1 | 6-12b-2-1 | 24 |
| 2 | 24 | Urethra | 20 Mar. 2017 | G2a-C4.S2 | 752614 | MK673384 | 6 | 12b | 2 | 1 | 6-12b-2-1 | 24 |
| 2 | 19 | Cervix | 4 Nov. 2016 | G2a-C5.S1 | 749039 | MK673385 | 100 | 16b | 2 | 1 | 100-16b-2-1 | 25 |
| 2 | 20 | Cervix | 12 Jul. 2017 | G2a-C5.S2 | 75150246 | MK673386 | 100 | 16a | 2 | 1 | 100-16a-2-1 | 26 |
| 1 | 44 | Rectum | 15 Nov. 2017 | G2a-C6.S1 | 78892902 | MK673387 | 7 | 11a | 2 | 1 | 7-11a-2-1 | 27 |
| 2 | 34 | Cervix | 18 Jan. 2018 | G2a-C6.S2 | 78893719 | MK673388 | 110 | 11a | 2 | 1 | 110-11a-2-1 | 28 |
| 1 | 37 | Urethra | 6 Feb. 2018 | G2b-C1.S1 | 78893872 | MK673389 | 2 | 11a | 3 | 1 | 2-11a-3-1 | 29 |
| 1 | 37 | Urethra | 26 Apr. 2018 | G2b-C1.S2 | 79897061 | MK673390 | 105 | 11a | 3 | 1 | 105-11a-3-1 | 30 |
| 1 | 24 | Urethra | 11 Apr. 2018 | G2b-C2.S1 | 97962396 | MK673391 | 105 | 9 | 3 | 1 | 105-09-3-1 | 31 |
| 1 | 24 | Urethra | 10 Oct. 2018 | G2b-C2.S2 | 97968446 | MK673392 | 105 | 9 | 3 | 1 | 105-09-3-1 | 31 |
| 1 | 40 | Urine | 10 Oct. 2018 | G3-C1a.S1 | 79890873 | MK673393 | 112 | 17b | 2 | 1 | 112-17b-2-1 | 19 |
| 1 | 38 | Urethra | 24 Oct. 2016 | G3-C2a.S1, G1-C18.S1 | 748733 | MK673394 | 118 | 12a | 2 | 1 | 118-12a-2-1 | 32 |
| 1 | 39 | Urethra | 4 Apr. 2017 | G3-C2a.S2, G1-C18.S2 | 752905 |  | NA | NA | NA | NA |  |  |
| 1 | 26 | Rectum | 12 Apr. 2017 | G3-C2b.S1 | 754058 | MK673395 | 107 | 10b | 2 | 1 | 107-10b-2-1 | 33 |
| 1 | 39 | Urethra | 4 Oct. 2017 | G3-C3b.S1 | 78892096 | MK673396 | 7 | 14a | 3 | 1 | 7-14a-3-1 | 3 |
| 2 | 43 | Cervix | 5 May 2017 | G3-C4a.S1 | 64553867 | MK673397 | 112 | 16a | 2 | 1 | 112-16a-2-1 | 34 |
| 1 | 41 | Urethra | 16 May 2017 | G3-C4b.S1 | 754528 | MK673398 | 112 | 16a | 2 | 1 | 112-16a-2-1 | 34 |
| 1 | 27 | Urethra | 15 May 2017 | G3-C5a.S1 | 754495 | MK673399 | 114 | 10a | 7 | 1 | 114-10a-7-1 | 35 |
| 2 | 22 | Vagina | 15 May 2017 | G3-C5b.S1 | 754498 | MK673400 | 114 | 10a | 7 | 1 | 114-10a-7-1 | 35 |
| 2 | 28 | Cervix | 31 Oct. 2017 | G3-C6a.S1 | 66390732 | MK673401 | 62 | 13a | 3 | 1 | 62-13a-3-1 | 36 |
| 1 | 28 | Urethra | 16 Nov. 2017 | G3-C6b.S1 | 78892923 | MK673402 | 62 | 13a | 3 | 1 | 62-13a-3-1 | 36 |
| 2 | 40 | Vagina | 20 Nov. 2017 | G3-C7a.S1 | 78892995 | MK673403 | 38 | 17a | 2 | 1 | 38-17a-2-1 | 37 |
| 1 | 49 | Urethra | 11 Jan. 2018 | G3-C7b.S1 | 79894224 | MK673404 | 38 | 17a | 2 | 1 | 38-17a-2-1 | 37 |
| 2 | 18 | Cervix | 24 Jan. 2018 | G3-C8a.S1 | 64769665 | MK673405 | 38 | 14b | 2 | 1 | 38-14b-2-1 | 38 |
| 1 | 21 | Urethra | 2 Feb. 2018 | G3-C8b.S1 | 79894624 | MK673406 | 38 | 15b | 2 | 1 | 38-15b-2-1 | 39 |
| 1 | 26 | Urethra | 10 May 2018 | G3-C9a.S1 | 79897353 | MK673407 | 113 | 12c | 3 | 1 | 113-12c-3-1 | 40 |
| 2 | 35 | Cervix | 11 May 2018 | G3-C9b.S1 | 62293024 | MK673408 | 120 | 10a | 2 | 1 | 120-10a-2-1 | 41 |
| 2 | 22 | Vagina | 8 Oct. 2018 | G3-C10b.S1 | 79890836 | MK673409 | 4 | 11b | 2 | 1 | 4-11b-2-1 | 18 |
| 1 | 30 | Urine | 7 Nov. 2018 | G3-C11a.S1 | 79892144 | MK673410 | 105 | 15a | 2 | 1 | 105-15a-2-1 | 42 |
| 2 | 28 | Pharinx | 14 Nov. 2018 | G3-C11b.S1 | 79892271 | MK673411 | 105 | 15a | 2 | 1 | 105-15a-2-1 | 42 |
| 1 | 25 | Urethra | 16 Nov. 2018 | G3-C11c.S1 | 79892346 | MK673412 | 105 | 14d | 2 | 1 | 105-14d-2-1 | 43 |
| 1 | 22 | Urethra | 19 Nov. 2018 | G3-C11d.S1 | 79892402 | MK673413 | 105 | 15a | 2 | 1 | 105-15a-2-1 | 42 |
| 1 | 42 | Urine | 3 Dec. 2018 | G3-C12a.S1 | 79892651 | MK673414 | 104 | 14b | 2 | 1 | 104-14b-2-1 | 44 |
| 2 | 45 | Vagina | 7 Dec. 2018 | G3-C12b.S1 | 79892739 | MK673415 | 104 | 14b | 2 | 1 | 104-14b-2-1 | 44 |
| 1 | 45 | Urethra | 10 Jan. 2014 | G4-C1 | 3133964 | MK673416 | 25 |  |  |  |  |  |
| 1 | 41 | Urethra | 3 Mar. 2014 | G4-C2 | 96046953 | MK673417 | 119 |  |  |  |  |  |
| 1 | 24 | Urethra | 13 May 2014 | G4-C3 | 749417 | MK673418 | 117 |  |  |  |  |  |
| 1 | 30 | Urethra | 22 Aug. 2014 | G4-C4 | 751218 | MK673419 | 115 |  |  |  |  |  |
| 1 | 27 | Urethra | 24 Oct. 2014 | G4-C5 | 752436 | MK673420 | 2 |  |  |  |  |  |
| 1 | 31 | Urethra | 26 Nov. 2014 | G4-C6 | 753102 | MK673421 | 2 |  |  |  |  |  |
| 1 | 35 | Urethra | 10 Feb. 2015 | G4-C7 | 62235395 | MK673422 | 7 |  |  |  |  |  |
| 1 | 33 | Urethra | 3 Jul. 2015 | G4-C8 | 757330 | MK673423 | 108 |  |  |  |  |  |
| 1 | 23 | Urine | 13 Mar. 2015 | G4-C9 | 755094 | MK673424 | 4 |  |  |  |  |  |
| 1 | 40 | Urethra | 1 Oct. 2015 | G4-C10 | 759365 | MK673425 | 6 |  |  |  |  |  |
| 1 | 21 | Urethra | 23 Dec. 2015 | G4-C11 | 741397 | MK673426 | 102 |  |  |  |  |  |
| 1 | 32 | Urethra | 7 Apr. 2016 | G4-C12 | 743705 | MK673427 | 70 |  |  |  |  |  |
| 1 | 24 | Urethra | 26 May 2016 | G4-C13 | 745092 | MK673428 | 2 |  |  |  |  |  |
| 1 | 30 | Urine | 30 Jun. 2016 | G4-C14 | 745567 | MK673429 | 51 |  |  |  |  |  |
| 2 | 35 | Cervix | 3 Aug. 2016 | G4-C15 | 746742 | MK673430 | 22 |  |  |  |  |  |
| 1 | 36 | Rectum | 10 Oct. 2016 | G4-C16 | 748544 | MK673431 | 51 |  |  |  |  |  |
| 2 | 26 | Cervix | 2 Dec. 2016 | G4-C17 | 749391 | MK673432 | 7 |  |  |  |  |  |
| 1 | 40 | Urine | 17 Jan. 2017 | G4-C18 | 750543 | MK673433 | 106 |  |  |  |  |  |
| 1 | 31 | Urethra | 3 Mar. 2017 | G4-C19 | 752356 | MK673434 | 7 |  |  |  |  |  |
| 1 | 44 | Urethra | 7 Apr. 2017 | G4-C20 | 364496 | MK673435 | 7 |  |  |  |  |  |
| 1 | 46 | Urethra | 15 May 2017 | G4-C21 | 754466 | MK673436 | 111 |  |  |  |  |  |
| 1 | 28 | Urethra | 21 Jul. 2017 | G4-C22 | 65705858 | MK673437 | 7 |  |  |  |  |  |
| 1 | 28 | Urethra | 20 Nov. 2017 | G4-C23 | 78892977 | MK673438 | 109 |  |  |  |  |  |
| 1 | 53 | Urine | 29 Aug. 2017 | G4-C24 | 79890955 | MK673439 | 116 |  |  |  |  |  |
| 2 | 28 | Cervix | 09 11 2017 | G4-C25 | 66391224 | MK673440 | 101 |  |  |  |  |  |
| 1 | 32 | Urine | 9 Jan. 2018 | G4-C26 | 79894149 | MK673441 | 62 |  |  |  |  |  |
| 1 | 47 | Rectum | 13 Mar. 2018 | G4-C27 | 79895187 | MK673442 | 105 |  |  |  |  |  |
| 2 | 18 | Vagina | 2 May 2018 | G4-C28 | 79897178 | MK673443 | 8 |  |  |  |  |  |
| 1 | 33 | Urine | 5 Jul. 2018 | G4-C29 | 79898256 | MK673444 | 2 |  |  |  |  |  |
| 2 | 41 | Cervix | 21 Oct. 2016 | G4-C30 | 97845974 | MK673445 | 110 |  |  |  |  |  |
| 1 | 25 | Urethra | 20 Apr. 2017 | G4-C31 | 754158 | MK673446 | 3 |  |  |  |  |  |

Sex: 1, male; 2, female. Groups: G1, first sample sensitive and second resistant to macrolides; G2, first and second samples both sensitive or both resistant; G3, couples/groups of sexual contacts; G4, not related and randomly distributed samples. GenBank accession numbers are those assigned to the corresponding adhesin gene fragment. For the genetic characterisation, a type number was assigned: for the MG191 marker of the *mgpB* adhesin gene, as a function of the previously described strains (Table S2, new genotypes obtained in this study were numbered from 99 to 120); for the MG309 marker of the lipoprotein gene, as a function of the variable numbers of short tandem repeats and their distribution (Figure S2); and for the internal transcribed spacers (ITS1 and ITS2) of the rRNA operon, in relation to single nucleotide polymorphisms (in ITS1, types 2 and 3 corresponded to two of the six sequence types assigned by Ma et al. [9] and type 7* carried the G1669A mutation in the 16S rRNA fragment; and in ITS2, type 1 corresponded to nucleotide A124 and type 2 to the G125A mutation). Each marker pattern resulting from the combination of these types was assigned a genetic profile or variant number. No amplification (NA) in these genes was obtained in one strain. In group 4, only the *mgpB* adhesin gene marker was studied.

*These strains were deposited in GenBank (accession numbers MN093118 and MN093119), and matched previously described strain LA197 (GenBank no. KC445178).
