# Supplementary material for: Molecular Typing of Mycoplasma genitalium-Positive Specimens Discriminates between Persistent and Recurrent Infections in Cases of Treatment Failure and Supports Contact Tracing
Source: Microorganisms. 2019 Nov 23;7(12):609. doi: 10.3390/microorganisms7120609 (PMC6955785; doi:10.3390/microorganisms7120609)
Supplement: Supplementary file 1 [file microorganisms-07-00609-s001.zip › microorganisms-621513-supple.zip!/2.TabS2.docx]

**Table S2.** Fasta sequences and MultAlin analysis (“Multiple sequence alignment with hierarchical clustering" F. Corpet, 1988, Nucl. Acids Res., 16 (22), 10881-10890) of the *mgpB* adhesin gene (MG191, 281 bp) with 121 strains previously deposited in GenBank (Horth et al. [4], Ma et al.[9], Musatovova & Baseman [10], Iverson et al. [11], Cazanave et al. [12], Chrisment et al. [13] and Pond et al. [14]) and the 105 strains described in this study (GenBank accession numbers MK673342 - MK673446). All the strains were aligned from a common start to a common end, corresponding to nucleotides 221774 - 222004 of the *Mycoplasma genitalium* reference strain G37 (accession number L43967). To make it easier to distinguish the different groups of strains (MultAlin check number), every other group is shaded in grey. *M. genitalium* G37 and MgPa were the same strain (genotype 1). Horth et al. [4] included genotypes 1-55 (numbered 1-55), genotype 9 having two different strains: DK16221[9] (9a) and M6285_R65[9] (9 b). Ma et al. [9] described genotypes 57-60 (accession numbers EU131379 - EU131382, numbered 57-60). Musatovova & Baseman [10] included 18 strains (accession numbers FJ750827 – FJ750844), FJ750833, FJ750839, FJ750838 and FJ750830 being respectively like genotypes 4, 5, 6 and 7 described by Hjorth et al. [4] (numbered 61-74 and 0 in the case of the previously described strains). Iverson et al. [11] included 9 strains (accession numbers DQ248087 – DQ248095), DQ248087 and DQ248095 being like genotype 2 and DQ248088 like genotype 24 (numbered 75-80 and 0 in the case of those previously described strains). Cazanave et al. [12] described genotypes 81-88 (accession numbers HM565865 – HM565872, numbered 81-88), and Chrisment et al. [13] genotypes 89-91 (accession numbers JQ439991 – JQ439993, numbered 89-91). Pond et al. [14] included 22 strains (accession numbers KC445139 – KC445160), 15 of them being previously described genotypes (numbered 92-98 and 0 in the case of those previously described). Genotypes 89, 90 and 91 assigned by Chrisment et al. [13] and Pond et al. [14] differed, and hence, they have been denoted as 89a, 90a and 91a, and 89b, 90b and 91b, respectively. In these seven studies, 101 different strains were described (numbered 1-98, genotype in brackets). In the present study, 45 out of the 105 *mgpB* gene sequences analysed differed from those previously described (in red) and 22 corresponding new genotypes were assigned from 99 to 120 (Table S1 and Figure S1).

* Strains FJ750844, DQ248090 and DQ248092 (numbered 74, 76 and 78) differed in some nucleotides not included in this alignment from genotypes 2, 6 and 3 described by Hjorth et al. [4], respectively.

Fasta sequences

>1.G37(L43967)

acttgaaacaataacaacttctcttcactaaagattactggagagaacccaggatcatttggattagtaagaagccaaaatgacaacttaaatatttcaagtgttacaaagaattctagtgatgataatctcaagtatctcaatgctgttgagaaataccttgatggtcagcaaaactttgcaatcagaaggtatgataacaacggtagagctttatatgatattaactta

>1.MgPa[1]

ACTTGAAACAATAACAACTTCTCTTCACTAAAGATTACTGGAGAGAACCCAGGATCATTTGGATTAGTAAGAAGCCAAAATGACAACTTAAATATTTCAAGTGTTACAAAGAATTCTAGTGATGATAATCTCAAGTATCTCAATGCTGTTGAGAAATACCTTGATGGTCAGCAAAACTTTGCAATCAGAAGGTATGATAACAACGGTAGAGCTTTATATGATATTAACTTA

>2.M2341[2]

ACTTGAAACAATAACAACTTCTCTTCACTAAAGATTACTGGAGAGAACCCAGGATCATTTGGATTAGTAAGAAGCCAAAATGACAACTTAAATATTTCAAGTGTTACAAAGAATGTTAGTGATGATAATCTCAAGTATCTCAATGCTGTTGAGAAATACCTTGATGGTCAGCAAAACTTTGCAATCAGAAGGTATGATAACAACGGTAGAGCTTTATATGATATTAACTTA

>3.H-p7791B11[3]

ACTTGAAACAATAACAACTTCTCTTCACTGAAGATTACTGGAGAGAACCCAGGATCATTTGGACTAGTAAGAAGCCAAAATGAGAACTTAAACATCGCAAGTGTTACAAAGAATGGTAGTGATGATAATCTCAAGTATCTTAATGCTGTTGAGAAGTACCTTGATGGTCAGCAAAACTTTGCAATCAGAAGGTATGATAACAACGGTAGAGCTTTATATGATATTAACTTA

>4.LF-p6486,L11[4]

ACTTGAAACAATAACAACTTCTCTTCACTGAAGATTACTGGAGAGAACCCAGGATCATTTGGACTAGTAAGAAGCCAAAATGAGAACTTAAACATCGCAAGTGTTACAAAGAATGATAGTGATGATAATCTCAAGTATCTTAATTCTGTTGAGAAATACCTTGATGGTCAGCAAAACTTTGCAATCAGAAGGTATGATAACAACGGTAGAGCTTTATATGATATTAACTTA

>5.LF-p6257,J11[5]

ACTTGAAACAATAACAACTTCTCTTCACTAAAGATTACTGGAGAGAACCCAGGATCATTTGGATTAGTAAGAAGCCAAAATGACAACTTAAATATTTCAAGTGTTACAAAGAATGTTAGTGATGATAATCTCAAGTATCTCAATGATGTTGAGAAATACCTTGATGGTCAGCAAAACTTTGCAATCAGAAGGTATGATAACAACGGTAGAGCTTTATATGATATTAACTTA

>6.DK_pr9[6]

ACTTGAAACAATAACAACTTCTCTTCACTAAAGATTACTGGAGAGAACCCAGGATCATTTGGATTAGTAAGAAGCCAAAATGACAACTTAAACATCGCAAGTGTTACAAAGAATGTTAGTGATGATAATCTCAAGTATCTCAATGCTGTTGAGAAATACCTTGATGGTCAGCAAAACTTTGCAATCAGAAGGTATGATAACAACGGTAGAGCTTTATATGATATTAACTTA

>7.M6271_E17-aus[7]

ACTTGAAACAATAACAACTTCTCTTCACTAAAGATTACTGGAGAGAACCCAGGATCATTTGGACTAGTAAGAAGCCAAAATGAGAACTTAAACATCGCAAGTGTTACAAAGAATGGTAGTGATGATAATCTCAAGTATCTCAATGCTGTTGAGAAATACCTTGATGGTCAGCAAAACTTTGCAATCAGAAGGTATGATAACAACGGTAGAGCTTTATATGATATTAACTTA

>8.66-0[8]

ACTTGAAACAATAACAACTTCTCTTCACTAAAGATTACTGGAGAGAACCCAGGATCATTTGGATTAGTAAGAAGCCAAAATGACAACTTAAATATTTCAAATGTTACAAAGAATGTTAGTGATGATAATCTCAAGTATCTCAATGATGTTGAGAAATACCTTGATGGTCAGCAAAACTTTGCAATCAGAAGGTATGATAACAACGGTAGAGCTTTATATGATATTAACTTA

>9a.DK16221[9]

ACTTGAAACAATAACAACTTCTCTTCACTAAAGATTACTGGAGAGAACCCAGGATCATTTGGATTAGTAAGAAGCCAAAATGACAACTTAAATATTTCAAGTGTTACAAAGAATGTTGTTACAAAGAATGTTAATGATGATAATCTCAAGTATCTCAATGCTGTTGAGAAATACCTTGATGGTCAGCAAAACTTTGCAATCAGAAGGTATGATAACAACGGTAGAGCTTTATATGATATTAACTTA

>9b.M6285_R65[9]

ACTTGAAACAATAACAACTTCTCTTCACTAAAGATTACTGGAGAGAACCCAGGATCATTTGGATTAGTAAGAAGCCAAAATGGCAACTTAAATATTTCAAGTGTTACAAAGAATGTTAGTGATGATAATCTCAAGTATCTCAATGATGTTGAGAAATACCTTGATGGTCAGCAAAACTTTGCAATCAGAAGGTATGATAACAACGGTAGAGCTTTATATGATATTAACTTA

>10.H7755g1[10]

ACTTGAAACAATAACAACTTCTCTTCACTAAAGATTACTGGAGAGAACCCAGGATCATTTGGATTAGTAAGAAGCCAAAATGACAACTTAAATATTTCAAGTGTTACAAAGAATGTTAGTGATGATAATCTCAAGTATCTCAATGCTGTTGAGAAATACCTTGATGATCGGCAAAACTTTGCAATCAGAAGGTATGATAACAACGGTAGAGCTTTATATGATATTAACTTA

>11.M2321[11]

ACTTGAAACAATAACAACTTCTCTTCACTAAAGATTACTGGAGAGAACCCAGGATCATTTGGATTAGTAAGAAGCCAAAATGACAACTTAAACATCGCAAGTGTTACAAAGAATGGTAGTGATGATAATCTCAAGTATCTCAATGCTGTTGAGAAATACCTTGATGGTCAGCAAAACTTTGCAATCAGAAGGTATGATAACAACGGTAGAGCTTTATATGATATTAACTTA

>12.H9504k1[12]

ACTTGAAACAATAACAACTTCTCTTCACTAAAGATTACTGGAGAGAACCCAGGATCATTTGGATTAGTAAGAAGCCAAAATGACAACTTAAATATTTCAAGTGTTACAAAGAATGTTAGTGATGATAATCTCAAGTATCTTAATAATGTTGAGAAGTACCTTAATGGTCAGCAAAACTTTGCAATCAGAAGGTATGATAACAACGGTAGAGCTTTATATGATATTAACTTA

>13.LF-p7523,O11[13]

ACTTGAAACAATAACAACTTCTCTTCACTGAAGATTACTGGAGAGAACCCAGGATCATTTGGACTAGTAAGAAGCCAAAATGAGAACTTAAACATCGCAAGTGTTACAAAGAATGGTAGTGATGATAATCTCAAGTATCTTAATGCTGTTGAGAAGTACCTTGATGGTAAGCAAAACTTTGCAATCAGAAGGTATGATAACAACGGTAGAGCTTTATATGATATTAACTTA

>14.M6286_R67[14]

ACTTGAAACAATAACAACTTCTCTTCACTGAAGATTACTGGAGAGAACCCAGGATCATTTGGACTAGTAAGAAGCCAAAATGAGAACTTAAACATCGCAAGTGTTACAAAGAATGATAGTGATGATAATCTCAAGTATCTCAATGCTGTTGAGAAATACCTTGATGGTCAGCAAAACTTTGCAATCAGAAGGTATGATAACAACGGTAGAGCTTTATATGATATTAACTTA

>15.DK523[15]

ACTTGAAACAATAACAACTTCTCTTCACTAAAGATTACTGGAGAGAACCCAGGATCATTTGGATTAGTAAGAAGCCAAAATGACAACTTAAATATTTCAAGTGTTACAAAGAATGTTAGTTATGATAATCTCAAGTATCTCAATGATGTTGAGAAATACCTTGATGGTCAGCAAAACTTTGCAATCAGAAGGTATGATAACAACGGTAGAGCTTTATATGATATTAACTTA

>16.LF-p6601,M11[16]

ACTTGAAACAATAACAACTTCTCTTCACTAAAGATTACTGGAGAGAACCCAGGATCATTTGGATTAGTAAGAAGCCAAAATGACAACTTAAATATTTCAAGTGTTACAAAGAATGTTGGTGATGATAATCTCAAGTATCTCAATGATGTTGAGAAATACCTTGATGGTCAGCAAAACTTTGCAATCAGAAGGTATGATAACAACGGTAGAGCTTTATATGATATTAACTTA

>17.RYH214[17]

ACTTGAAACAATAACAACTTCTCTTCACTAAAGATTACTGGAGAGAACCCAGGATCATTTGGATTAGTAAGAAGCCAAAATGACAACTTAAATATTTCAAGTGCTACAAAGAATGTTAGTGATGATAATCTCAAGTATCTCAATGATGTTGAGAAATACCTTGATGGTCAGCAAAACTTTGCAATCAGAAGGTATGATAACAACGGTAGAGCTTTATATGATATTAACTTA

>18.76-0[18]

ACTTGAAACAATAACAACTTCTCTTCACTAAAGATTACTGGAGAGAACCCAGGATCATTTGGATTAGTAAGAAGCCAAAATGACAACTTAAATATTTCAAGTGTTACAAAGAATGTTAGTGATGATAATCTCAAGTATCTCAATTATGTTGAGAAATACCTTGATGGTCAGCAAAACTTTGCAATCAGAAGGTATGATAACAACGGTAGAGCTTTATATGATATTAACTTA

>19.LF-p7262,P11[19]

ACTTGAAACAATAACAACTTCTCTTCACTAAAGATTACTGGAGAGAACCCAGGATCATTTGGATTAGTAAGAAGCCAAAATGACAACTTAAATATTTCAAATGTTACAAAGAATGTTAGTGATGATAATCTCAAGTATCTCAATGCTGTTGAGAAATACCTTGATGGTCAGCAAAACTTTGCAATCAGAAGGTATGATAACAACGGTAGAGCTTTATATGATATTAACTTA

>20.M2300[20]

ACTTGAAACAATAACAACTTCTCTTCACTAAAGATTACTGGAGAGAACCCAGGATCATTTGGATTAGTAAGAAGCCAAAATGACAACTTAAATATTTCAAGTGTTACAAAGAATGTTAGTTATGATAATCTCAAGTATCTCAATGCTGTTGAGAAATACCTTGATGGTCAGCAAAACTTTGCAATCAGAAGGTATGATAACAACGGTAGAGCTTTATATGATATTAACTTA

>21.2094_Ume[21]

ACTTGAAACAATAACAACTTCTCTTCACTAAAGATTACTGGAGAGAACCCAGGATCATTTGGATTAGTAAGAAGCCAAAATGACAACTTAAATATTTCAAGTGTTACAAAGAATGTTAGTGATGATAATCTCAAGTATCTCAATACTGTTGAGAAATACCTTGATGGTCAGCAAAACTTTGCAATCAGAAGGTATGATAACAACGGTAGAGCTTTATATGATATTAACTTA

>22.314F_rus[22]

ACTTGAAACAATAACAACTTCTCTTCACTAAAGATTACTGGAGAGAACCCAGGATCATTTGGATTAGTAAGAAGCCAAAATGACAACTTAAATATTTCAAGTGTTACAAAGAATGTTGGTGATGATAATCTCAAGTATCTCAATGCTGTTGAGAAATACCTTGATGGTCAGCAAAACTTTGCAATCAGAAGGTATGATAACAACGGTAGAGCTTTATATGATATTAACTTA

>23.45/01-UK_preg[23]

ACTTGAAACAATAACAACTTCTCTTCACTAAAGATTACTGGAGAGAACCCAGGATCATTTGGATTAGTAAGAAGCCAAAATGACAACTTAAATATTTCAAGTGTTACAAAGAGTGTTAGTGATGATAATCTCAAGTATCTCAATGCTGTTGAGAAATACCTTGATGGTCAGCAAAACTTTGCAATCAGAAGGTATGATAACAACGGTAGAGCTTTATATGATATTAACTTA

>24.5336_D[24]

ACTTGAAACAATAACAACTTCTCTTCACTAAAGATTACTGGAGAGAACCCAGGATCATTTGGATTAGTAAGAAGCCAAAATGACAACTTAAATATTTCAAATGTTACAAAGAATGTTAGTAATGATAATCTCAAGTATCTCAATGATGTTGAGAAATACCTTGATGGTCAGCAAAACTTTGCAATCAGAAGGTATGATAACAACGGTAGAGCTTTATATGATATTAACTTA

>25.RYH217[25]

ACTTGAAACAATAACAACTTCTCTTCACTAAAGATTACTGGAGAGAACCCAGGATCATTTGGATTAGTAAGAAGCCAAAATGACAACTTAAATATTTCAAGTGTTACAAAGAATGTTAGTGATGATAATCTCAAGTATCTCAATGCTGTTGAGAAATACCTTGATGATCAGCAAAACTTTGCAATCAGAAGGTATGATAACAACGGTAGAGCTTTATATGATATTAACTTA

>26.RYH117[26]

ACTTGAAACAATAACAACTTCTCTTCACTAAAGATTACTGGAGAGAACCCAGGATCATTTGGATTAGTAAGAAGCCAAAATGACAACTTAAATATTTTAAGTGTTACAAAGAATTTTAGTGATGATAATCTCAAGTATCTCAATGCTGTTGAGAAATACCTTGATGGTCAGCAAAACTTTGCAATCAGAAGGTATGATAACAACGGTAGAGCTTTATATGATATTAACTTA

>27.35M_rus[27]

ACTTGAAACAATAACAACTTCTCTTCACTAAAAATTACTGGAGAGAACCCAGGATCATTTGGATTAGTAAGAAGCCAAAATGACAACTTAAATATTTCAAGTGTTACAAAGAATATTAGTGATGATAATCTCAAGTATCTCAATGCTGTTGAGAAATACCTTGATGGTCAGCAAAACTTTGCAATCAGAAGGTATGATAACAACGGTAGAGCTTTATATGATATTAACTTA

>28.M6270_D24-aus[28]

ACTTGAAACAATAACAACTTCTCTTCACTAAAGATTACTGGAGAGAACCCAGGATCATTTGGATTAGTAAGAAGCCAAAATGACAACTTAAATATTTCAAGTGCTACAAAGAATGTTAATGATGATAATCTCAAGTATCTCAATGCTGTTGAGAAATACCTTGATGGTCAGCAAAACTTTGCAATCAGAAGGTATGATAACAACGGTAGAGCTTTATATGATATTAACTTA

>29.1-0[29]

ACTTGAAACAATAACAACTTCTCTTCACTAAAGATTACTGGAGAGAACCCAGGATCATTTGGATTAGTAAGAAGCCAAAATGACAACTTAAATATTTCAAATGTTACAAAGAATGTTGGTGATAATAATCTCAAGTATCTCAATGATGTTGAGAAATACCTTGATGGTCAGCAAAACTTTGCAATCAGAAGGTATGATAACAACGGTAGAGCTTTATATGATATTAACTTA

>30.H346j1[30]

ACTTGAAACAATAACAACTTCTCTTCACTAAAGATTACTGGAGAGAACCCAGGATCATTTGGATTAGTAAGAAGCCAAAATGACAACTTAAACATCGCAAGTGTTACAAAGAATGTTGGTGATGATAATCTCAAGTATCTCAATGCTGTTGAGAAATACCTTGATGGTCAGCAAAACTTTGCAATCAGAAGGTATGATAACAACGGTAGAGCTTTATATGATATTAACTTA

>31.DK15776[31]

ACTTGAAACAATAACAACTTCTCTTCACTAAAGATTACTGGAGAGAACCCAGGATCATTTGGATTAGTAAGAAGCCAAAATGACAACTTAAACATGGCAAGTGTTACAAAGAATGGTAGTGATGATAATCTCAAGTATCTCAATGCTGTTGAGAAATACCTTGATGGTCAGCAAAACTTTGCAATCAGAAGGTATGATAACAACGGTAGAGCTTTATATGATATTAACTTA

>32.M2288[32]

ACTTGAAACAATAACAACTTCTCTTCACTAAAGATTACTGGAGAGAACCCAGGATCATTTGGATTAGTAAGAAGCCAAAATGACAACTTAAATATTTCAAGTGTTACAAAGAATCCTAATGATGATAATCTCGAGTATCTCAATGCTGTTGAGAAATACCTTGATGGTCAGCAAAACTTTGCAATCAGAAGGTATGATAACAACGGTAGAGCTTTATATGATATTAACTTA

>33.M6328_R66[33]

ACTTGAAACAATAACAACTTCTCTTCACTAAAGATTACTGGAGAGAACCCAGGATCATTTGGATTAGTAAGAAGCCAAAATGACAACTTAAATATTTCAAGTGTTACAAAGAATGTTAATTATGATTATCTCGATGCTGTTGAGAAATACCTTGATGGTCGGCAAAACTTTGCAATCAGAAGGTATGATAACAACGGTAGAGCTTTATATGATATTAACTTA

>34.19-0[34]

ACTTGAAACAATAACAACTTCTCTTCACTAAAGATTACTGGAGAGAACCCAGGATCATTTGGATTAGTAAGAAGCCAAAATGACAACTTAAATATTTCAAGTGTTACAAAGAATGATGCTATTGATGATAATCTCAAGTATCTTAATGATGTTGAGAAGTACCTTAATGGTCAGCAAAACTTTGCAATCAGAAGGTATGATAACAACGGTAGAGCTTTATATGATATTAACTTA

>35.RYH70[35]

ACTTGAAACAATAACAACTTCTCTTCACTAAAGATTACTGGAGAGAACCCAGGATCATTTGGATTAGTAAGAAGCCAAAATGACAACTTAAATATTTCAAATGTTACAAAGAGTGTTACTGATGATAATCTCAAGTATCTCAATGATGTTGAGAAATACCTTGATGATAAGCAAAACTTTGCAATCAGAAGGTATGATAACAACGGTAGAGCTTTATATGATATTAACTTA

>36.1182_Ume[36]

ACTTGAAACAATAACAACTTCTCTTCACTAAAGATTACTGGAGAGAACCCAGGATCATTTGGATTAGTAAGAAGCCAAAATGACAACTTAAATATTTCAAGTGTTATAAAGAGTACTAGTGATGATAATCTCAAGTATCTCAATGCTGTTAAGGAATACCTTGATGGTCAGCAAAACTTTGCAATCAGAAGGTATGATAACAACGGTAGAGCTTTATATGATATTAACTTA

>37.H12202o1[37]

ACTTGAAACAATAACAACTTCTCTTCACTAAAGATTACTGGAGAGAACCCAGGATCATTTGGATTAGTAAGAAGCCAAAATGACAACTTAAATATTCCAAGTATTACAAAGGGGGTTAGTAATGATAATCTCAAGTATCTCAATACTGTTGAGAAATACCTTGATGGTCAGCAAAACTTTGCAATCAGAAGGTATGATAACAACGGTAGAGCTTTATATGATATTAACTTA

>38.N4219[38]

ACTTGAAACAATAACAACTTCTCTTCACTGAAGATTACTGGAGAGAACCCAGGATCATTTGGACTAGTAAGAAGCCAAAATGAGAACTTAAACATCGCAAGTGTTACAAAGAATGATAATGATGATAATCTCAAGTATCTTAATTCTGTTGAGAAATACCTTGATGGTCAGCAAAACTTTGCAATCAGAAGGTATGATAACAACGGTAGAGCTTTATATGATATTAACTTA

>39.129-0[39]

ACTTGAAACAATAACAACTTCTCTTCACTGAAGATTACTGGAGAGAACCCAGGATCATTTGGACTAGTAAGAAGCCAAAATGAGAACTTAAACATCGCAAGTGTTACAAAGAATGATAGTGATGATAATCTCAAGTATCTTAATTCTGTTGAGAAATACCTTGATGATCAGCAAAACTTTGCAATCAGAAGGTATGATAACAACGGTAGAGCTTTATATGATATTAACTTA

>40.RYH23[40]

ACTTGAAACAATAACAACTTCTCTTCACTGAAGATTACTGGAGAGAACCCAGGATCATTTGGACTAGTAAGAAGCCAAAATGAGAACTTAAACATCAAAAGTGTTACAAAGAATGATAGTGATGATAATCTCAAGTATCTTAATTCTGTTGAGAAATACCTTGATGATCAGCAAAACTTTGCAATCAGAAGGTATGATAACAACGGTAGAGCTTTATATGATATTAACTTA

>41.RYH115[41]

ACTTGAAACAATAACAACTTCTCTTCACTGAAGATTACTGGAGAGAACCCAGGATCATTTGGACTAGTAAGAAGCCAAAATGAGAACTTAAACATCGCAAGTGTTACAAAGAATAATAGTAATGATAATCTCGAGTATCTTAATTCTGTTGAGAAATACCTTGATGGTCAGCAAAACTTTGCAATCAGAAGGTATGATAACAACGGTAGAGCTTTATATGATATTAACTTA

>42.H12755v1[42]

ACTTGAAACAATAACAACTTCTCTTCACTGAAGATTACTGGAGAGAACCCAGGATCATTTGGACTAGTAAAAAGCCAAAATGAGAACTTAAACATCGCAAGTGTTACAAAGAATGGTAGTGATGATAATCTCAAGTATCTTAATGCTGTTGAGAAGTACCTTGATGGTCAGCAAAACTTTGCAATCAGAAGGTATGATAACAACGGTAGAGCTTTATATGATATTAACTTA

>43.123-0[43]

ACTTGAAACAATAACAACTTCTCTTCACTGAAGATTACTGGAGAGAACCCAGGATCATTTGGACTAGTAAGAAGCCAAAATGAGAACTTAAACATCGCAAGTGTTACAAAGAATGGTAGTGATGATAATCTCAAGTATCTTAATTCTGTTGAGAAGTACCTTGATGGTCAGCAAAACTTTGCAATCAGAAGGTATGATAACAACGGTAGAGCTTTATATGATATTAACTTA

>44.168-0[44]

ACTTGAAACAATAACAACTTCTCTTCACTGAAGATTACTGGAGAGAACCCAGGATCATTTGGACTAGTAAGAAGCCAAAATGAGAACTTAAACATCGCAAGTGTTACAAAGAATGGTAGTGATGATAATCTCAAGTATCTTAATGATGTTGAGAAGTACCTTGATGGTCAGCAAAACTTTGCAATCAGAAGGTATGATAACAACGGTAGAGCTTTATATGATATTAACTTA

>45.399F_rus[45]

ACTTGAAACAATAACAACTTCTCTTCACTGAAGATTACTGGAGAGAACCCAGGATCATTTGGACTAGTAAGAAGCCAAAATGAGAACTTAAACATCGCAAGTGTTACAAAGAATGGTAGTGATGATAATCTCAAGTATCTTAATGCTGTTGAGAAGTACCTTGATGGTCAACAAAACTTTGCAATCAGAAGGTATGATAACAACGGTAGAGCTTTATATGATATTAACTTA

>46.RYH181[46]

ACTTGAAACAATAACAACTTCTCTTCACTGAAGATTACTGGAGAGAACCCAGGATCATTTGGACTAGTAAGAAGCCAAAATGAGAACTTAAACATCGCAAGTGTTACAAAGAATGGTAGTGATGATAATCTCAAGTATCTTAGTGCTGTTGAGAAGTACCTTGATGGTCAGCAAAACTTTGCAATCAGAAGGTATGATAACAACGGTAGAGCTTTATATGATATTAACTTA

>47.18-0R[47]

ACTTGAAACAATAACAACTTCTCTTCACTGAAGATTACTGGAGAGAACCCAGGATCATTTGGACTAGTAAGAAGCCAAAATGAGAACTTAAACATCGCAAGTGTTACAAAGAATGGTAATGATGATAATCTCAAGTATCTTAATGCTGTTGAGAAGTACCTTGATGGTCAGCAAAACTTTGCAATCAGAAGGTATGATAACAACGGTAGAGCTTTATATGATATTAACTTA

>48.Taqman3672[48]

ACTTGAAACAATAACAACTTCTCTTCACTGAAGATTACTGGAGAGAACCCAGGATCATTTGGACTAGTAAGAAGCCAAAATGACAACTTAAACATCGCAAGTGTTACAAAGAATGGTAGTGATGATAATCTCAAGTATCTTAATGCTGTTGAGAAGTACCTTGATGGTAAGCAAAACTTTGCAATCAGAAGGTATGATAACAACGGTAGAGCTTTATATGATATTAACTTA

>49.DK11844[49]

ACTTGAAACAATAACAACTTCTCTTCACTGAAGATTACTGGAGAGAACCCAGGATCATTTGGATTAGTAAGAAGCCAAAATGAGAACTTAAACATCGCAAGTGTTACAAAGAATGGTAGTGATGATAATCTCGAGTATCTTAATGCTGTTGAGAAATACCTTGATGGTCAGCAAAACTTTGCAATCAGAAGGTATGATAACAACGGTAGAGCTTTATATGATATTAACTTA

>50.LF-p7551,S11[50]

ACTTGAAACAATAACAACTTCTCTTCACTAAAGATTACTGGAGAGAACCCAGGATCATTTGGATTAGTAAGAAGTCAAAATGAGAACTCAAACATCGCAAGTGTTACAAAGAATGATAGTGATGATAATCTCAAGTATCTTAATTCTGTTGAGAAATACCTTGATGGTCAGCAAAACTTTGCAATCAGAAGGTATGATAACAACGGTAGAGCTTTATATGATATTAACTTA

>51.111-0[51]

ACTTGAAACAATAACAACTTCTCTTCACTGAAGATTACTGGAGAGAACCCAGGATCATTTGGATTAGTAAGAAGCCAAAATGCGAACTTAAATATCGCAAGTGTTCAAAAGCAATCAGGTGATGATAATCTCAAGTATCTTAATGCTGTTGAGAAATACCTTGATGGTCAGCAAAACTTTGCAATCAGAAGGTATGATAACAACGGTAGAGCTTTATATGATATTAACTTA

>52.H13051f1[52]

ACTTGAAACAATAACAACTTCTCTTCACTGAAGATTACTGGAGAGAACCCAGGATCATTTGGATTAGTAAGAAGCCAAAATGCGAACTTAAATATCGCAAGTGTTCAAAAGCAATCAGGTGATGATAATCTCAAGTATCTTAATTCTGTTGAGAAATACCTTAATGGTCAGCAAAACTTTGCAATCAGAAGGTATGATAACAACGGTAGAGCTTTATATGATATTAACTTA

>53.H-p9300A191[53]

ACTTGAAACAATAACAACTTCTCTTCACTAAAGATTACTGGAGAGAACCCAGGATCATTTGGATTAGTAAGAAGCCAAAATGACAACTTAAATATTTCAAGTGTTACAAAGAATGCTAATGATGATAATCTCAAGTATCTCAATGCTGTTGAGAAATACCTTGATGGTCAGCAAAACTTTGCAATCAGAAGGTATGATAACAACGGTAGAGCTTTATATGATATTAACTTA

>54.H97050g2[54]

ACTTGAAACAATAACAACTTCTCTTCACTGAAGATTACTGGAGAGAACCCAGGATCATTTGGACTAGTAAGAAGCCAAAATGAGAACTTAAAAATCGTAAGTGTTACAAAGAATGATAGTGATGATAATCTCAAGTATCTTAATTCTGTTGAGAAATACCTTGATGATCAGCAAAACTTTGCAATCAGAAGGTATGATAACAACGGTAGAGCTTTATATGATATTAACTTA

>55.H8692[55]

ACTTGAAACAATAACAACTTCTCTTCACTGAAGATTACTGGAGAGAATCCAGGATCATTTGGACTAGTAAGAAGCCAAAATGAGAACTTAAACATCGCAAGTGTTACAAAGAATGATAATAATGATAATCTCAAGTATCTTAATGCTGTTGAGAAGTACCTTGATGGTCAGCAAAACTTTGCAATCAGAAGGTATGATAACAACGGTAGAGCTTTATATGATATTAACTTA

>57.EU131379

acttgaaacaataacaacttctcttcactgaagattactggagagaacccaggatcatttggactagtaagaagccaaaatgagaacttaaacatcgcaagtgttacaaagaatggtaataatgataatctcaagtatcttaatgctgttgagaagtaccttgatggtaagcaaaactttgcaatcagaaggtatgataacaacggtagagctttatatgatattaactta

>58.EU131380

acttgaaacaataacaacttctcttcactgaagattactggagagaacccaggatcatttggattagtaagaagccaaaatgcgaacttaaatatcgcaagtgttcaaaagcaatcaggtgatgataatctcaagtatcttgatgctgttgagaaatgccttgatgatcagcaaaactttgcaatcagaaggtatgataacaacggtagagctttatatgatattaactta

>59.EU131381

acttgaaacaataacaacttctcttcactaaagattactggagagaacccaggatcatttggattagtaagaagccaaaatgcgaacttaaatatcgcaagtgttcaaaagcaatcaggtgatgataatctcaagtatcttaatgctgttgagaaataccttgatggtcagcaaaactttgcaatcagaaggtatgataacaacggtagagctttatatgatattaactta

>60.EU131382

acttgaaacaataacaacttctcttcactgaagattactggagagaacccaggatcatttggactagtaagaaaccaaaatgagaacttaaacatcgcaagtgttacaaagaatggtagtgatgataatctcaagtatcttaatgctgttgagaagtaccttgatggtcagcaaaactttgcaatcagaaggtatgataacaacggtagagctttatatgatattaactta

>61.FJ750827

acttgaaacaataacaacttctcttcactaaagattactggagagaacccaggatcatttggattagtaagaagccaaaatgacaacttaaatatttcaagtgttacaaagaatgttagtgatgataatctcaagtatcttaattctgttgagaaataccttgatggtcagcaaaactttgcaatcagaaggtatgataacaacggtagagctttatatgatattaactta

>62.FJ750828

acttgaaacaataacaacttctcttcactaaagattactggagagaacccaggatcatttggactagtaagaagccaaaatgagaacttaaacatcgcaagtgttacaaagaatgatagtgatgataatctcaagtatcttaattctgttgagaaataccttgatggtcagcaaaactttgcaatcagaaggtatgataacaacggtagagctttatatgatattaactta

>63.FJ750829

acttgaaacaataacaacttctcttcactgaagattactggagagaacccaggatcatttggactagtaagaagccaaaatgagaacttaaacatcgcaagtgttacaaagaatggtagtgatgataatctcaagtatctcaatgctgttgagaaataccttgatggtcagcaaaactttgcaatcagaaggtatgataacaacggtagagctttatatgatattaactta

>0.FJ750830[7]

acttgaaacaataacaacttctcttcactaaagattactggagagaacccaggatcatttggactagtaagaagccaaaatgagaacttaaacatcgcaagtgttacaaagaatggtagtgatgataatctcaagtatctcaatgctgttgagaaataccttgatggtcagcaaaactttgcaatcagaaggtatgataacaacggtagagctttatatgatattaactta

>64.FJ750831

acttgaaacaataacaacttctcttccctgaagattactggagagaacccaggatcatttggactagtaagaagccaaaatgagaacttaaacatcgaaagtgttacaaagaatgatagtgatgataatctcaagtatcttaattctgttgagaaataccttgatggtcagcaaaactttgcaatcagaaggtatgataacaacggtagagctttatatgatattaactta

>65.FJ750832

acttgaaacaataacaacttctcttcactaaagattactggagagaacccaggatcatttggattagtaagaagccaaaatgacaacttaaacatcgcaagtgttacaaagaatgctagtgatgataatctcaagtatctcaatgctgttgagaaataccttgatggtcagcaaaactttgcaatcagaaggtatgataacaacggtagagctttatatgatattaactta

>0.FJ750833[4]

acttgaaacaataacaacttctcttcactgaagattactggagagaacccaggatcatttggactagtaagaagccaaaatgagaacttaaacatcgcaagtgttacaaagaatgatagtgatgataatctcaagtatcttaattctgttgagaaataccttgatggtcagcaaaactttgcaatcagaaggtatgataacaacggtagagctttatatgatattaactta

>66.FJ750834

acttgaaacaataacaacttctcttcactaaagattactggagagaacccaggatcatttggattagtaagaagccaaaatgacaacttaaatatttcaagtgttacaaaggatgttagtgatgataatctcaagtatctcaatgctgttgagaaataccttgatggtcagcaaaactttgcaatcagaaggtatgataacaacggtagagctttatatgatattaactta

>67.FJ750835

acttgaaacaataacaacttctcttcactgaagattactggagagaatccaggatcatttggattagtaagaagccaaaatgcgaacttaaatatcgcaagtgttcaaaagcaatcaggtgatgataatctcaagtatcttaatgctgttgagaaataccttgatggtcagcaaaactttgcaatcagaaggtatgataacaacggtagagctttatatgatattaactta

>68.FJ750836

acttgaaacaataacaacttctcttcactaaagattactggagagaacccaggatcatttggattagtaagaagccaaaatgacaacttaaatatttcaagtgttacagttagtggtgataatctcaagtatctcaatgctgttgagaaataccttgatggtcagcaaaactttgcaatcagaaggtatgataacaacggtagagctttatatgatattaactta

>69.FJ750837

acttgaaacaataacaacttctcttcactaaagattactggagagaacccaggatcatttggattagtaagaagccaaaatgacaacttaaatatttcaagtgttacaagtggtgataatctcaagtatctcaatgctgttgagaaataccttgatggtcagcaaaactttgcaatcagaaggtatgataacaacggtagagctttatatgatattaactta

>0.FJ750838[6]

acttgaaacaataacaacttctcttcactaaagattactggagagaacccaggatcatttggattagtaagaagccaaaatgacaacttaaacatcgcaagtgttacaaagaatgttagtgatgataatctcaagtatctcaatgctgttgagaaataccttgatggtcagcaaaactttgcaatcagaaggtatgataacaacggtagagctttatatgatattaactta

>0.FJ750839[5]

acttgaaacaataacaacttctcttcactaaagattactggagagaacccaggatcatttggattagtaagaagccaaaatgacaacttaaatatttcaagtgttacaaagaatgttagtgatgataatctcaagtatctcaatgatgttgagaaataccttgatggtcagcaaaactttgcaatcagaaggtatgataacaacggtagagctttatatgatattaactta

>70.FJ750840

acttgaaacaataacaacttctcttcactaaagattactggagagaacccaggatcatttggattagtaagaagccaaaatgacaacttaaatatttcaaatgttacaaagaatgttagtgatgataatctcaagtatctcaattatgttgagaaataccttgatgatcagcaaaactttgcaatcagaaggtatgataacaacggtagagctttatatgatattaactta

>71.FJ750841

acttgaaacaataacaacttctcttcactaaagattactggagagaacccaggatcatttggattagtaagaagccaaaatgacaacttaaatatttcaagtgttacagataaggttagtcatgataatctcatgtatctcaatgctgttgagaaataccttgatgatcagcaaaactttgcaatcagaaggtatgataacaacggtagagctttatatgatattaactta

>72.FJ750842

acttgaaacaataacaacttctcttcactgaagattactggagagaatccaggatcatttggattagtaagaagccaaaatgcgaacttaaatatcgcaagtgttcaaaagcaatctggtgatgataatctcaagtatcttaatgctgttgagaaataccttgatggtcagcaaaactttgcaatcagaaggtatgataacaacggtagagctttatatgacattaactta

>73.FJ750843

acttgaaacaataacaacttctcttcactaaagattactggagagaacccaggatcatttggattagtaagaagccaaaatgacaacttaaatatttcaagtgttacaaagaatgctgattatgataatctcaagtatcttaataatgttgagaagtaccttaatggtcagcaaaactttgcaatcagaaggtatgataacaacggtagagctttatatgatattaactta

>74.FJ750844

acttgaaacaataacaacttctcttcactaaagattactggagagaacccaggatcatttggattagtaagaagccaaaatgacaacttaaatatttcaagtgttacaaagaatgttagtgatgataatctcaagtatctcaatgctgttgagaaataccttgatggtcagcaaaactttgcaatcagaaggtatgataacaacggtagagctttatatgatattaactta

>0.DQ248087[2]

acttgaaacaataacaacttctcttcactaaagattactggagagaacccaggatcatttggattagtaagaagccaaaatgacaacttaaatatttcaagtgttacaaagaatgttagtgatgataatctcaagtatctcaatgctgttgagaaataccttgatggtcagcaaaactttgcaatcagaaggtatgataacaacggtagagctttatatgatattaactta

>0.DQ248088[24]

acttgaaacaataacaacttctcttcactaaagattactggagagaacccaggatcatttggattagtaagaagccaaaatgacaacttaaatatttcaaatgttacaaagaatgttagtaatgataatctcaagtatctcaatgatgttgagaaataccttgatggtcagcaaaactttgcaatcagaaggtatgataacaacggtagagctttatatgatattaactta

>75.DQ248089

acttgaaacaataacaacttctcttcactgaagattactggagagaacccaggatcatttggactagtaagaagccaaaatgagaacttaaacatcgcaagtgttacaaagcatagtagtgatgataatctcaagtatcttaatgctgttgagaagtaccttgatggtcagcaaaactttgcaatcagaaggtatgataacaacggtagagctttatatgatattaactta

>76.DQ248090

acttgaaacaataacaacttctcttcactaaagattactggagagaacccaggatcatttggattagtaagaagccaaaatgacaacttaaacatcgcaagtgttacaaagaatgttagtgatgataatctcaagtatctcaatgctgttgagaaataccttgatggtcagcaaaactttgcaatcagaaggtatgataacaacggtagagctttatatgatattaactta

>77.DQ248091

acttgaaacaataacaacttctcttcactaaagattactggagagaacccaggatcatttggattagtaagaagccaaaatgacaacttaaacatcgcaagtgctacaaagaatgttaatgatgataatctcaagtatctcaatactgttgagaaataccttgatggtcagcaaaactttgcaatcagaaggtatgataacaacggtagagctttatatgatattaactta

>78.DQ248092

acttgaaacaataacaacttctcttcactgaagattactggagagaacccaggatcatttggactagtaagaagccaaaatgagaacttaaacatcgcaagtgttacaaagaatggtagtgatgataatctcaagtatcttaatgctgttgagaagtaccttgatggtcagcaaaactttgcaatcagaaggtatgataacaacggtagagctttatatgatattaactta

>79.DQ248093

acttgaaacaataacaacttctcttcactaaagattactggagagaacccaggatcatttggattagtaagaagccaaaatgacaacttaaatatttcaagtgttacaaagaatgttagtgatgataatctcaagtatctcaataatgttgagaaataccttgatggtcagcaaaactttgcaatcagaaggtatgataacaacggtagagctttatatgatattaactta

>80.DQ248094

acttgaaacaataacaacttctcttcactgaagattactggagagaacccaggatcatttggactagtaagaagccaaaatgagaacttaaacatcgcaagtgttacaaagcatggtagtgatgataatctcaagtatcttaatgctgttgagaagtaccttgatggtcagcaaaactttgcaatcagaaggtatgataacaacggtagagctttatatgatattaactta

>0.DQ248095[2]

acttgaaacaataacaacttctcttcactaaagattactggagagaacccaggatcatttggattagtaagaagccaaaatgacaacttaaatatttcaagtgttacaaagaatgttagtgatgataatctcaagtatctcaatgctgttgagaaataccttgatggtcagcaaaactttgcaatcagaaggtatgataacaacggtagagctttatatgatattaactta

>81.HM565865[81]

acttgaaacaataacaacttctcttcactaaagattactggagagaacccaggatcatttggattagtaagaagccaaaatgacaacttaaatatttcaagtgttataaagaatgttagtgatgataatctcaagtatctcaatgctgttgagaaataccttgatggtcagcaaaactttgcaatcagaaggtatgataacaacggtagagctttatatgatattaactta

>82.HM565866[82]

acttgaaacaataacaacttctcttcactaaagattactggagagaacccaggatcatttggattagtaagaagccaaaatgacaacttaaatatttcaagtgttgcaaagaatgttagtgatgataatctcaagtatctcaatgctgttgagaaataccttgatggtcagcaaaactttgcaatcagaaggtatgataacaacggtagagctttatatgatattaactta

>83.HM565867[83]

acttgaaacaataacaacttctcttcactaaagattactggagagaacccaggatcatttggattagtaagaagccaaaatgacaacttaaatatttcaagtgttacaaagaatgttaatgatgataatctcaagtatctcaatactgttgagaaataccttgatggtcagcaaaactttgcaatcagaaggtatgataacaacggtagagctttatatgatattaactta

>84.HM565868[84]

acttgaaacaataacaacttctcttcactgaagattactggagagaacccaggatcatttggattagtaagaagccaaaatgcgaacttaaatatcgcaaatgttcaaaagcaatcaggtgatgataatctcaagtatcttaattctgttgagaaataccttgatggtcagcaaaactttgcaatcagaaggtatgataacaacggtagagctttatatgatattaactta

>85.HM565869[85]

acttgaaacaataacaacttctcttcactgaagattactggagagaacccaggatcatttggactagtaagaagccaaaatgagaacttaaacatcgcaagtgttacaaagaatgatagtgttgataatctcaagtatcttaattctgttgagaaataccttgatggtcagcaaaactttgcaatcagaaggtatgataacaacggtagagctttatatgatattaactta

>86.HM565870[86]

acttgaaacaataacaacttctcttcactaaagattactggagagaacccaggatcatttggattagtaagaagccaaaatgacaacttaaatatttcaagtgttacaaagaatgttggggatgataatctcaagtatctcaatgctgttgagaaataccttgatggtcagcaaaactttgcaatcagaaggtatgataacaacggtagagctttatatgatattaactta

>87.HM565871[87]

acttgaaacaataacaacttctcttcactgaagattactggagagaacccaggatcatttggactagtaagaagccaaaatgacaacttaaatatttcaagtgttacaaaggatggtaatgatgataatctcaagtatcttaatactgttgagaagtaccttgatggtcagcaaaactttgcaatcagaaggtatgataacaacggtagagctttatatgatattaactta

>88.HM565872[88]

acttgaaacaataacaacttctcttcactaaagattactggagagaacccaggatcatttggattagtaagaagccaaaatgacaacttaaatatttcaagtgttacaaagaatgttagtgatgataatttcaagtatctcaatgatgttgagaaataccttgatggtcagcaaaactttgcaatcagaaggtatgataacaacggtagagctttatatgatattaactta

>89.JQ439991[89a]

acttgaaacaataacaacttctcttcactaaagattactggagagaacccaggatcatttggattagtaagaagccaaaatgacaacttaaatatttcaagtgttacaaagaatgttaatgataatctcaagtatctcaatgctgttgagaaataccttgatggtcagcaaaactttgcaatcagaaggtatgataacaacggtagagctttatatgatattaactta

>90.JQ439992[90a]

acttgaaacaataacaacttctcttcactaaagattactggagagaacccaggatcatttggattagtaagaagccaaaatgacaacttaaacatcgcaagtgttacaaaggatgttagtgatgataatctcaagtatctcaatgctgttgagaaataccttgatggtcagcaaaactttgcaatcagaaggtatgataacaacggtagagctttatatgatattaactta

>91.JQ439993[91a]

Acttgaaacaataacaacttctcttcactgaagattactggagagaacccaggatcatttggactagtaagaagccaaaatgagaacttaaacatcgcaagtgttacaaagaatgatgatgatgataatctcaagtatcttaattctgttgagaaataccttgatggtcagcaaaactttgcaatcagaaggtatgataacaacggtagagctttatatgatattaactta

>0.KC445139[3]

acttgaaacaataacaacttctcttcactgaagattactggagagaacccaggatcatttggactagtaagaagccaaaatgagaacttaaacatcgcaagtgttacaaagaatggtagtgatgataatctcaagtatcttaatgctgttgagaagtaccttgatggtcagcaaaactttgcaatcagaaggtatgataacaacggtagagctttatatgatattaactta

>0.KC445140[89b]

acttgaaacaataacaacttctcttcactaaagattactggagagaacccaggatcatttggactagtaagaagccaaaatgagaacttaaacatcgcaagtgttacaaagaatggtagtgatgataatctcaagtatctcaatgctgttgagaaataccttgatggtcagcaaaactttgcaattagaaggtatgataacaacggtagagctttatatgatattaactta

>0.KC445141[3]

acttgaaacaataacaacttctcttcactgaagattactggagagaacccaggatcatttggactagtaagaagccaaaatgagaacttaaacatcgcaagtgttacaaagaatggtagtgatgataatctcaagtatcttaatgctgttgagaagtaccttgatggtcagcaaaactttgcaatcagaaggtatgataacaacggtagagctttatatgatattaactta

>0.KC445142[90b]

acttgaaacaataacaacttctcttcactaaagattactggagagaacccaggatcatttggattagtaagaagtcaaaatgacaacttaaatatttcaagtgttacaaagaatgttagtgatgataatctcaagtatctcaatgctgttgagaaataccttgatggtcagcaaaactttgcaatcagaaggtatgataacaacggtagagctttatatgatattaactta

>0.KC445143[91b]

acttgaaacaataacaacttctcttcactaaagattactggagagaacccaggatcatttggattagtaagaagccaaaatgacaacttaaatatttcaaatgttacaaagaatgttagtgatgataatctcaagtatctcaataatcttgagaaataccttgatggtcagcaaaactttgcaatcagaaggtatgataacaacggtagagctttatatgatattaactta

>0.KC445144[21]

acttgaaacaataacaacttctcttcactaaagattactggagagaacccaggatcatttggattagtaagaagccaaaatgacaacttaaatatttcaagtgttacaaagaatgttagtgatgataatctcaagtatctcaatactgttgagaaataccttgatggtcagcaaaactttgcaatcagaaggtatgataacaacggtagagctttatatgatattaactta

>92.KC445145[92]

acttgaaacaataacaacttctcttcactgaagattactggagagaacccaggatcatttggactagtaagaagccaaaatgagaacttaaacatcgcaagtgttacaaagaatgttagtgatgataatctcaagtatcttaatgctgttgagaagtaccttgatggtcagcaaaactttgcaatcagaaggtatgataacaacggtagagctttatatgatattaactta

>0.KC445146[8]

acttgaaacaataacaacttctcttcactaaagattactggagagaacccaggatcatttggattagtaagaagccaaaatgacaacttaaatatttcaaatgttacaaagaatgttagtgatgataatctcaagtatctcaatgatgttgagaaataccttgatggtcagcaaaactttgcaatcagaaggtatgataacaacggtagagctttatatgatattaactta

>93.KC445147[93]

acttgaaacaataacaacttctcttcactaaagattactggagagaacccaggatcatttggattagtaagaagccaaaatgacagcttaaatatttcaaatgttacaaagaatgttagtgatgataatctcaagtatctcaatgatgttgagaaataccttgatggtcggcaaaactttgcaatcagaaggtatgataacaacggtagagctttatatgatattaactta

>0.KC445148[2]

acttgaaacaataacaacttctcttcactaaagattactggagagaacccaggatcatttggattagtaagaagccaaaatgacaacttaaatatttcaagtgttacaaagaatgttagtgatgataatctcaagtatctcaatgctgttgagaaataccttgatggtcagcaaaactttgcaatcagaaggtatgataacaacggtagagctttatatgatattaactta

>0.KC445149[4]

acttgaaacaataacaacttctcttcactgaagattactggagagaacccaggatcatttggactagtaagaagccaaaatgagaacttaaacatcgcaagtgttacaaagaatgatagtgatgataatctcaagtatcttaattctgttgagaaataccttgatggtcagcaaaactttgcaatcagaaggtatgataacaacggtagagctttatatgatattaactta

>0.KC445150[90b]

acttgaaacaataacaacttctcttcactaaagattactggagagaacccaggatcatttggattagtaagaagtcaaaatgacaacttaaatatttcaagtgttacaaagaatgttagtgatgataatctcaagtatctcaatgctgttgagaaataccttgatggtcagcaaaactttgcaatcagaaggtatgataacaacggtagagctttatatgatattaactta

>94.KC445151[94]

acttgaaacaataacaacttctcttcactaaagattactggagagaacccaggatcatttggactagtaagaagccaaaatgagaacttaaacatcgcaagtgttacaaagaatggtagtgatgataatctcaagtatctcaatgctgttgagaaataccttgatgatcagcaaaactttgcaatcagaaggtatgataacaacggtagagctttatatgatattaactta

>95.KC445152[95]

acttgaaacaataacaacttctcttcactaaagattactggagagaacccaggatcatttggattagtaagaagccaaaatgacaacttaaatatttcaagtgttacaaagaatgctagtgatgataatctcaagtatctcaatgctgttgagaaataccttgatggtcagcaaaactttgcaatcagaaggtatgataacaacggtagagctttatatgatattaactta

>0.KC445153[4]

acttgaaacaataacaacttctcttcactgaagattactggagagaacccaggatcatttggactagtaagaagccaaaatgagaacttaaacatcgcaagtgttacaaagaatgatagtgatgataatctcaagtatcttaattctgttgagaaataccttgatggtcagcaaaactttgcaatcagaaggtatgataacaacggtagagctttatatgatattaactta

>96.KC445154[96]

acttgaaacaataacaacttctcttcactgaagattactggagagaacccaggatcatttggactagtaagaagccaaaatgacaacttaaacatcgcaagtgttaaagagaataatagtgatgataatctcaagtatcttaattctgttgagaaataccttgatggtcagcaaaactttgcaatcagaaggtatgataacaacggtagagctttatatgatattaactta

>97.KC445155[97]

acttgaaacaataacaacttctcttcactaaagattactggagagaacccaggatcatttggactagtaagaagccaaaatgagaacttaaacatcgcaagtgttacaaagaatggtagtgatgataatctcaagtatctcaatgctgttgagaaatacctttatggtcagcaaaactttgcaatcagaaggtatgataacaacggtagagctttatatgatattaactta

>0.KC445156[24]

acttgaaacaataacaacttctcttcactaaagattactggagagaacccaggatcatttggattagtaagaagccaaaatgacaacttaaatatttcaaatgttacaaagaatgttagtaatgataatctcaagtatctcaatgatgttgagaaataccttgatggtcagcaaaactttgcaatcagaaggtatgataacaacggtagagctttatatgatattaactta

>0.KC445157[44]

acttgaaacaataacaacttctcttcactgaagattactggagagaacccaggatcatttggactagtaagaagccaaaatgagaacttaaacatcgcaagtgttacaaagaatggtagtgatgataatctcaagtatcttaatgatgttgagaagtaccttgatggtcagcaaaactttgcaatcagaaggtatgataacaacggtagagctttatatgatattaactta

>0.KC445158[23]

acttgaaacaataacaacttctcttcactaaagattactggagagaacccaggatcatttggattagtaagaagccaaaatgacaacttaaatatttcaagtgttacaaagagtgttagtgatgataatctcaagtatctcaatgctgttgagaaataccttgatggtcagcaaaactttgcaatcagaaggtatgataacaacggtagagctttatatgatattaactta

>98.KC445159[98]

acttgaaacaataacaacttctcttcactaaagattactggagagaacccaggatcatttggattagtaagaagccaaaatgacaacttaaacatcgcaagtgttacaaaaaatggcagtgatgataatctcaagtatcttaataatgttgagaagtaccttaatggtcagcaaaactttgcaatcagaaggtatgataacaacggtagagctttatatgatattaactta

>0.KC445160[3]

acttgaaacaataacaacttctcttcactgaagattactggagagaacccaggatcatttggactagtaagaagccaaaatgagaacttaaacatcgcaagtgttacaaagaatggtagtgatgataatctcaagtatcttaatgctgttgagaagtaccttgatggtcagcaaaactttgcaatcagaaggtatgataacaacggtagagctttatatgatattaactta

>MK673342

ACTTGAAACAATAACAACTTCTCTTCACTAAAGATTACTGGAGAGAACCCAGGATCATTTGGATTAGTAAGAAGCCAAAATGACAACTTAAATATTTCAAGTGTTACAAAGAATGTTAGTGATGATAATCTCAAGTATCTCAATGCTGTTGAGAAATACCTTGATGGTCAGCAAAACTTTGCAATCAGAAGGTATGATAACAACGGTAGAGCTTTATATGATATTAACTTA

>MK673343

ACTTGAAACAATAACAACTTCTCTTCACTAAAGATTACTGGAGAGAACCCAGGATCATTTGGATTAGTAAGAAGCCAAAATGACAACTTAAATATTTCAAGTGTTACAAAGAATGTTAGTGATGATAATCTCAAGTATCTCAATGCTGTTGAGAAATACCTTGATGGTCAGCAAAACTTTGCAATCAGAAGGTATGATAACAACGGTAGAGCTTTATATGATATTAACTTA

>MK673344

ACTTGAAACAATAACAACTTCTCTTCACTAAAGATTACTGGAGAGAACCCAGGATCATTTGGATTAGTAAGAAGCCAAAATGACAACTTAAATATTTCAAGTGTTACAAAGAATGTTGGTGATGATAATCTCAAGTATCTCAATGCTGTTGAGAAATACCTTGATGGTCAGCAAAACTTTGCAATCAGAAGGTATGATAACAACGGTAGAGCTTTATATGATATTAACTTA

>MK673345

ACTTGAAACAATAACAACTTCTCTTCACTAAAGATTACTGGAGAGAACCCAGGATCATTTGGATTAGTAAGAAGCCAAAATGACAACTTAAATATTTCAAGTGTTACAAAGAATGTTGGTGATGATAATCTCAAGTATCTCAATGCTGTTGAGAAATACCTTGATGGTCAGCAAAACTTTGCAATCAGAAGGTATGATAACAACGGTAGAGCTTTATATGATATTAACTTA

>MK673346

ACTTGAAACAATAACAACTTCTCTTCACTAAAGATTACTGGAGAGAACCCAGGATCATTTGGACTAGTAAGAAGCCAAAATGAGAACTTAAACATCGCAAGTGTTACAAAGAATGGTAGTGATGATAATCTCAAGTATCTCAATGCTGTTGAGAAATACCTTGATGGTCAGCAAAACTTTGCAATCAGAAGGTATGATAACAACGGTAGAGCTTTATATGATATTAACTTA

>MK673347

ACTTGAAACAATAACAACTTCTCTTCACTAAAGATTACTGGAGAGAACCCAGGATCATTTGGACTAGTAAGAAGCCAAAATGAGAACTTAAACATCGCAAGTGTTACAAAGAATGGTAGTGATGATAATCTCAAGTATCTCAATGCTGTTGAGAAATACCTTGATGGTCAGCAAAACTTTGCAATCAGAAGGTATGATAACAACGGTAGAGCTTTATATGATATTAACTTA

>MK673348

ACTTGAAACAATAACAACTTCTCTTCACTAAAGATTACTGGAGAGAACCCAGGATCATTTGGATTAGTAAGAAGCCAAAATGACAACTTAAACATCGCAAGTGTTACAAAGAATGTTAGTGATGATAATCTCAAGTATCTCAATGCTGTTGAGAAATACCTTGATGGTCAGCAAAACTTTGCAATCAGAAGGTATGATAACAACGGTAGAGCTTTATATGATATTAACTTA

>MK673349

ACTTGAAACAATAACAACTTCTCTTCACTAAAGATTACTGGAGAGAACCCAGGATCATTTGGATTAGTAAGAAGCCAAAATGACAACTTAAACATCGCAAGTGTTACAAAGAATGTTAGTGATGATAATCTCAAGTATCTCAATGCTGTTGAGAAATACCTTGATGGTCAGCAAAACTTTGCAATCAGAAGGTATGATAACAACGGTAGAGCTTTATATGATATTAACTTA

>MK673350

ACTTGAAACAATAACAACTTCTCTTCACTGAAGATTACTGGAGAGAACCCAGGATCATTTGGACTAGTAAGAAGCCAAAATGAGAACTTAAACATCGCAAGTGTTACAAAGAATGATAGTGATGATAATcTCAAGTATCTTAATTCTGTTGAGAAATACCTTGATGGTCAGCAAAACTTTGCAATCAGAAGGTATGATAACAACGGTAGAGCTTTATATGATATTAACTTA

>MK673351

ACTTGAAACAATAACAACTTCTCTTCACTGAAGATTACTGGAGAGAACCCAGGATCATTTGGACTAGTAAGAAGCCAAAATGAGAACTTAAACATCGCAAGTGTTACAAAGAATGATAGTGATGATAATCTCAAGTATCTTAATTCTGTTGAGAAATACCTTGATGGTCAGCAAAACTTTGCAATCAGAAGGTATGATAACAACGGTAGAGCTTTATATGATATTAACTTA

>MK673352

ACTTGAAACAATAACAACTTCTCTTCACTAAAGATTACTGGAGAGAACCCAGGATCATTTGGATTAGTAAGAAGCCAAAATGACAACTTAAATATTTCAAATGTTATTACAAAGAATAATGTTAGTGATGATAATCTCAAGTATCTCAATGATGTTGAGAAATACCTTGATGGTCAGCAAAACTTTGCAATCAGAAGGTATGATAACAACGGTAGAGCTTTATATGATATTAACTTA

>MK673353

ACTTGAAACAATAACAACTTCTCTTCACTAAAGATTACTGGAGAGAACCCAGGATCATTTGGATTAGTAAGAAGCCAAAATGACAACTTAAATATTTCAAATGTTATTACAAAGAATAATGTTAGTGATGATAATCTCAAGTATCTCAATGATGTTGAGAAATACCTTGATGGTCAGCAAAACTTTGCAATCAGAAGGTATGATAACAACGGTAGAGCTTTATATGATATTAACTTA

>MK673354

ACTTGAAACAATAACAACTTCTCTTCACTAAAGATTACTGGAGAGAACCCAGGATCATTTGGACTAGTAAGAAGCCAAAATGAGAACTTAAACATCGCAAGTGTTACAAAGAATGGTAGTGATGATAATCTCAAGTATCTCAATGCTGTTGAGAAATACCTTGATGGTCAGCAAAACTTTGCAATCAGAAGGTATGATAACAACGGTAGAGCTTTATATGATATTAACTTA

>MK673355

ACTTGAAACAATAACAACTTCTCTTCACTAAAGATTACTGGAGAGAACCCAGGATCATTTGGACTAGTAAGAAGCCAAAATGAGAACTTAAACATCGCAAGTGTTACAAAGAATGGTAGTGATGATAATCTCAAGTATCTCAATGCTGTTGAGAAATACCTTGATGGTCAGCAAAACTTTGCAATCAGAAGGTATGATAACAACGGTAGAGCTTTATATGATATTAACTTA

>MK673356

ACTTGAAACAATAACAACTTCTCTTCACTAAAGATTACTGGAGAGAACCCAGGATCATTTGGACTAGTAAGAAGCCAAAATGAGAACTTAAACATCGCAAGTGTTACAAAGAATGGTAGTGATGATAATCTCAAGTATCTCAATGCTGTTGAGAAATACCTTGATGGTCAGCAAAACTTTGCAATCAGAAGGTATGATAACAACGGTAGAGCTTTATATGATATTAACTTA

>MK673357

ACTTGAAACAATAACAACTTCTCTTCACTAAAGATTACTGGAGAGAACCCAGGATCATTTGGATTAGTAAGAAGCCAAAATGACAACTTAAACATCGCAAGTGTTACAAAGAATGTTAGTGATGATAATCTCAAGTATCTCAATGCTGTTGAGAAATACCTTGATGGCCAGCAAAACTTTGCAATCAGAAGGTATGATAACAACGGTAGAGCTTTATATGATATTAACTTA

>MK673358

ACTTGAAACAATAACAACTTCTCTTCACTAAAGATTACTGGAGAGAACCCAGGATCATTTGGATTAGTAAGAAGCCAAAATGACAACTTAAACATCGCAAGTGTTACAAAGAATGTTAGTGATGATAATCTCAAGTATCTCAATGCTGTTGAGAAATACCTTGATGGCCAGCAAAACTTTGCAATCAGAAGGTATGATAACAACGGTAGAGCTTTATATGATATTAACTTA

>MK673359

ACTTGAAACAATAACAACTTCTCTTCACTAAAGATTACTGGAGAGAACCCAGGATCATTTGGATTAGTAAGAAGCCAAAATGACAACTTAAATATTTCAAGTGTTACAAAGAATGTTAGTGATGATAATCTCAAGTATCTCAATGCTGTTGAGAAATACCTTGATGGTCAGCAAAACTTTGCAATCAGAAGGTATGATAACAACGGTAGAGCTTTATATGATATTAACTTA

>MK673360

acttgaaacaataacaacttctcttcactaaagattactggagagaacccaggatcatttggattagtaagaagccaaaatgacaacttaaatatttcaagtgttacaaagaatgttagtgatgataatctcaagtatctcaatgctgttgagaaataccttgatggtcagcaaaactttgcaatcagaaggtatgataacaacggtagagctttatatgatattaactta

>MK673361

ACTTGAAACAATAACAACTTCTCTTCACTAAAGATTACTGGAGAGAACCCAGGATCATTTGGATTAGTAAGAAGCCAAAATGACAACTTAAACATCGCAAGTGTTACAAAGAATGTTAGTGATGATAATCTCAAGTATCTCAATGCTGTTGAGAAATACCTTGATGGTCAGCAAAACTTTGCAATCAGAAGGTATGATAACAACGGTAGAGCTTTATATGATATTAACTTA

>MK673362

ACTTGAAACAATAACAACTTCTCTTCACTAAAGATTACTGGAGAGAACCCAGGATCATTTGGATTAGTAAGAAGCCAAAATGACAACTTAAACATCGCAAGTGTTACAAAGAATGTTAGTGATGATAATCTCAAGTATCTCAATGCTGTTGAGAAATACCTTGATGGTCAGCAAAACTTTGCAATCAGAAGGTATGATAACAACGGTAGAGCTTTATATGATATTAACTTA

>MK673363

ACTTGAAACAATAACAACTTCTCTTCACTAAAGATTACTGGAGAGAACCCAGGATCATTTGGATTAGTAAGAAGCCAAAATGACAACTTAAATATTTCAAGTGTTACAAAGAATGTTAGTGATGATAATCTCAAGTATCTCAATGCTGTTGAGAAATACCTTGATGGTCAGCAAAACTTTGCAATCAGAAGGTATGATAACAACGGTAGAGCTTTATATGATATTAACTTA

>MK673364

ACTTGAAACAATAACAACTTCTCTTCACTAAAGATTACTGGAGAGAACCCAGGATCATTTGGATTAGTAAGAAGCCAAAATGACAACTTAAATATTTCAAGTGTTACAAAGAATGTTAGTGATGATAATCTCAAGTATCTCAATGCTGTTGAGAAATACCTTGATGGTCAGCAAAACTTTGCAATCAGAAGGTATGATAACAACGGTAGAGCTTTATATGATATTAACTTA

>MK673365

ACTTGAAACAATAACAACTTCTCTTCACTAAAGATTACTGGAGAGAACCCAGGATCATTTGGATTAGTAAGAAGCCAAAATGACAACTTAAACATCGCAAGTGTTACAAAGAATGGTAGTGATGATAATCTCAAGTATCTCAATGCTGTTGAGAAATACCTTGATGGTCAGCAAAACTTTGCAATCAGAAGGTATGATAACAACGGTAGAGCTTTATATGATATTAACTTA

>MK673366

ACTTGAAACAATAACAACTTCTCTTCACTAAAGATTACTGGAGAGAACCCAGGATCATTTGGATTAGTAAGAAGCCAAAATGACAACTTAAACATCGCAAGTGTTACAAAGAATGGTAGTGATGATAATCTCAAGTATCTCAATGCTGTTGAGAAATACCTTGATGGTCAGCAAAACTTTGCAATCAGAAGGTATGATAACAACGGTAGAGCTTTATATGATATTAACTTA

>MK673367

ACTTGAAACAATAACAACTTCTCTTCACTGAAGATTACTGGAGAGAACCCAGGATCATTTGGACTAGTAAGAAGCCAAAATGAGAACTTAAACATCGCAAGTGTTACAAAGAATGATAGTGATGATAATCTCAAGTATCTTAATTCTGTTGAGAAATACCTTGATGGTCAGCAAAACTTTGCAATCAGAAGGTATGATAACAACGGTAGAGCTTTATATGATATTAACTTA

>MK673368

ACTTGAAACAATAACAACTTCTCTTCACTGAAGATTACTGGAGAGAACCCAGGATCATTTGGACTAGTAAGAAGCCAAAATGAGAACTTAAACATCGCAAGTGTTACAAAGAATGATAGTGATGATAATCTCAAGTATCTTAATTCTGTTGAGAAATACCTTGATGGTCAGCAAAACTTTGCAATCAGAAGGTATGATAACAACGGTAGAGCTTTATATGATATTAACTTA

>MK673369

ACTTGAAACAATAACAACTTCTCTTCACTAAAGATTACTGGAGAGAACCCAGGATCATTTGGACTAGTAAGAAGCCAAAATGAGAACTTAAACATCGCAAGTGTTACAAAGAATGGTAGTGATGATAATCTCAAGTATCTCAATGCTGTTGAGAAATACCTTGATGGTCAGCAAAACTTTGCAATCAGAAGGTATGATAACAACGGTAGAGCTTTATATGATATTAACTTA

>MK673370

ACTTGAAACAATAACAACTTCTCTTCACTAAAGATTACTGGAGAGAACCCAGGATCATTTGGACTAGTAAGAAGCCAAAATGAGAACTTAAACATCGCAAGTGTTACAAAGAATGGTAGTGATGATAATCTCAAGTATCTCAATGCTGTTGAGAAATACCTTGATGGTCAGCAAAACTTTGCAATCAGAAGGTATGATAACAACGGTAGAGCTTTATATGATATTAACTTA

>MK673371

ACTTGAAACAATAACAACTTCTCTTCACTGAAGATTACTGGAGAGAACCCAGGATCATTTGGACTAGTAAGAAGCCAAAATGAGAACTTAAACATCGCAAGTGTTACAAAGAATGGTAGTGATGATAATCTCAAGTATCTTAATGCTGTTGAGAAATACCTTGATGGTCAGCAAAACTTTGCAATCAGAAGGTATGATAACAACGGTAGAGCTTTATATGATATTAACTTA

>MK673372

ACTTGAAACAATAACAACTTCTCTTCACTGAAGATTACTGGAGAGAACCCAGGATCATTTGGACTAGTAAGAAGCCAAAATGAGAACTTAAACATCGCAAGTGTTACAAAGAATGGTAGTGATGATAATCTCAAGTATCTTAATGCTGTTGAGAAATACCTTGATGGTCAGCAAAACTTTGCAATCAGAAGGTATGATAACAACGGTAGAGCTTTATATGATATTAACTTA

>MK673373

ACTTGAAACAATAACAACTTCTCTTCACTGAAGATTACTGGAGAGAACCCAGGATCATTTGGACTAGTAAGAAGCCAAAATGAGAACTTAAACATCGCAAGTGTTACAAAGAATGATAGTGATGATAATCTCAAGTATCTTAATTCTGTTGAGAAATACCTTGATGGTCAGCAAAACTTTGCAATCAGAAGGTATGATAACAACGGTAGAGCTTTATATGATATTAACTTA

>MK673374

ACTTGAAACAATAACAACTTCTCTTCACTGAAGATTACTGGAGAGAACCCAGGATCATTTGGACTAGTAAGAAGCCAAAATGAGAACTTAAACATCGCAAGTGTTACAAAGAATGATAGTGATGATAATCTCAAGTATCTTAATTCTGTTGAGAAATACCTTGATGGTCAGCAAAACTTTGCAATCAGAAGGTATGATAACAACGGTAGAGCTTTATATGATATTAACTTA

>MK673375

ACTTGAAACAATAACAACTTCTCTTCACTAAAGATTACTGGAGAGAACCCAGGATCATTTGGATTAGTAAGAAGCCAAAATGACAACTTAAATATTTCAAATGTTATTACAAAGAATGATGCTAATGATGATAATCTCAAGTATCTCAATGATGTTGAGAAATACCTTGATGGTCAGCAAAACTTTGCAATCAGAAGGTATGATAACAACGGTAGAGCTTTATATGATATTAACTTA

>MK673376

ACTTGAAACAATAACAACTTCTCTTCACTAAAGATTACTGGAGAGAACCCAGGATCATTTGGATTAGTAAGAAGCCAAAATGACAACTTAAATATTTCAAATGTTATTACAAAGAATGATGCTAATGATGATAATCTCAAGTATCTCAATGATGTTGAGAAATACCTTGATGGTCAGCAAAACTTTGCAATCAGAAGGTATGATAACAACGGTAGAGCTTTATATGATATTAACTTA

>MK673377

ACTTGAAACAATAACAACTTCTCTTCACTAAAGATTACTGGAGAGAACCCAGGATCATTTGGATTAGTAAGAAGCCAAAATGACAACTTAAATATTTCAAATGTTACAAAGAATGTTAGTAATGATAATCTCAACTATCTCAATGATGTTGAGAAATACCTTGATGGTCAGCAAAACTTTGCAATCAGAAGGTATGATAACAACGGTAGAGCTTTATATGATATTAACTTA

>MK673378

ACTTGAAACAATAACAACTTCTCTTCACTAAAGATTACTGGAGAGAACCCAGGATCATTTGGATTAGTAAGAAGCCAAAATGACAACTTAAATATTTCAAATGTTACAAAGAATGTTAGTAATGATAATCTCAACTATCTCAATGATGTTGAGAAATACCTTGATGGTCAGCAAAACTTTGCAATCAGAAGGTATGATAACAACGGTAGAGCTTTATATGATATTAACTTA

>MK673379

ACTTGAAACAATAACAACTTCTCTTCACTAAAGATTACTGGAGAGAACCCAGGATCATTTGGATTAGTAAGAAGCCAAAATGACAACTTAAATATTTCAAGTGTTACAAAGAATGTTAGTGATGATAATCTCAAGTATCTCAATGCTGTTGAGAAATACCTTGATGGTCAACAAAACTTTGCAATCAGAAGGTATGATAACAACGGTAGAGCTTTATATGATATTAACTTA

>MK673380

ACTTGAAACAATAACAACTTCTCTTCACTGAAGATTACTGGAGAGAACCCAGGATCATTTGGACTAGTAAGAAGCCAAAATGAGAACTTAAACATCGCAAGTGTTACAAAGAATGGTAGTGATGATAATCTCAAGTATCTTAATGCTGTTGAGAAATACCTTGATGGTCAGCAAAACTTTGCAATCAGAAGGTATGATAACAACGGTAGAGCTTTATATGATATTAACTTA

>MK673381

ACTTGAAACAATAACAACTTCTCTTCACTGAAGATTACTGGAGAGAACCCAGGATCATTTGGACTAGTAAGAAGCCAAAATGAGAACTTAAACATCGCAAGTGTTACAAAGAATGATAGTGATGATAATCTCAAGTATCTTAATTCTGTTGAGAAATACCTTGATGGTCAGCAAAACTTTGCAATCAGAAGGTATGATAACAACGGTAGAGCTTTATATGATATTAACTTA

>MK673382

ACTTGAAACAATAACAACTTCTCTTCACTGAAGATTACTGGAGAGAACCCAGGATCATTTGGACTAGTAAGAAGCCAAAATGAGAACTTAAACATCGCAAGTGTTACAAAGAATGATAGTGATGATAATCTCAAGTATCTTAATTCTGTTGAGAAATACCTTGATGGTCAGCAAAACTTTGCAATCAGAAGGTATGATAACAACGGTAGAGCTTTATATGATATTAACTTA

>MK673383

ACTTGAAACAATAACAACTTCTCTTCACTAAAGATTACTGGAGAGAACCCAGGATCATTTGGATTAGTAAGAAGCCAAAATGACAACTTAAACATCGCAAGTGTTACAAAGAATGTTAGTGATGATAATCTCAAGTATCTCAATGCTGTTGAGAAATACCTTGATGGTCAGCAAAACTTTGCAATCAGAAGGTATGATAACAACGGTAGAGCTTTATATGATATTAACTTA

>MK673384

ACTTGAAACAATAACAACTTCTCTTCACTAAAGATTACTGGAGAGAACCCAGGATCATTTGGATTAGTAAGAAGCCAAAATGACAACTTAAACATCGCAAGTGTTACAAAGAATGTTAGTGATGATAATCTCAAGTATCTCAATGCTGTTGAGAAATACCTTGATGGTCAGCAAAACTTTGCAATCAGAAGGTATGATAACAACGGTAGAGCTTTATATGATATTAACTTA

>MK673385

ACTTGAAACAATAACAACTTCTCTTCACTAAAGATTACTGGAGAGAACCCAGGATCATTTGGATTAGTAAGAAGCCAAAATGACAACTTAAACATCGCAAGTGTTACAAAGAATGTTAGTGATGATAATCTCAAGTATCTCAAGGCTGTTGAGAAATACCTTGATGGTCAGCAAAACTTTGCAATCAGAAGGTATGATAACAACGGTAGAGCTTTATATGATATTAACTTA

>MK673386

ACTTGAAACAATAACAACTTCTCTTCACTAAAGATTACTGGAGAGAACCCAGGATCATTTGGATTAGTAAGAAGCCAAAATGACAACTTAAACATCGCAAGTGTTACAAAGAATGTTAGTGATGATAATCTCAAGTATCTCAAGGCTGTTGAGAAATACCTTGATGGTCAGCAAAACTTTGCAATCAGAAGGTATGATAACAACGGTAGAGCTTTATATGATATTAACTTA

>MK673387

ACTTGAAACAATAACAACTTCTCTTCACTAAAGATTACTGGAGAGAACCCAGGATCATTTGGACTAGTAAGAAGCCAAAATGAGAACTTAAACATCGCAAGTGTTACAAAGAATGGTAGTGATGATAATCTCAAGTATCTCAATGCTGTTGAGAAATACCTTGATGGTCAGCAAAACTTTGCAATCAGAAGGTATGATAACAACGGTAGAGCTTTATATGATATTAACTTA

>MK673388

ACTTGAAACAATAACAACTTCTCTTCACTAAAGATTACTGGAGAGAACCCAGGATCATTTGGATTAGTAAGAAGCCAAAATGACAACTTAAATATTTCAAATGTTATTACAAAGAATAATGTTAGTGATGATAATCTCAAGTATCTCAATGATGTTGAGAAATACCTTGATGGTCAGCAAAACTTTGCAATCAGAAGGTATGATAACAACGGTAGAGCTTTATATGATATTAACTTA

>MK673389

ACTTGAAACAATAACAACTTCTCTTCACTAAAGATTACTGGAGAGAACCCAGGATCATTTGGATTAGTAAGAAGCCAAAATGACAACTTAAATATTTCAAGTGTTACAAAGAATGTTAGTGATGATAATCTCAAGTATCTCAATGCTGTTGAGAAATACCTTGATGGTCAGCAAAACTTTGCAATCAGAAGGTATGATAACAACGGTAGAGCTTTATATGATATTAACTTA

>MK673390

ACTTGAAACAATAACAACTTCTCTTCACTGAAGATTACTGGAGAGAACCCAGGATCATTTGGACTAGTAAGAAGCCAAAATGAGAACTTAAACATCGCAAGTGTTACAAAGAATGGTAGTGATGATAATCTCAAGTATCTTAATGCTGTTGAGAAATACCTTGATGGTCAGCAAAACTTTGCAATCAGAAGGTATGATAACAACGGTAGAGCTTTATATGATATTAACTTA

>MK673391

ACTTGAAACAATAACAACTTCTCTTCACTGAAGATTACTGGAGAGAACCCAGGATCATTTGGACTAGTAAGAAGCCAAAATGAGAACTTAAACATCGCAAGTGTTACAAAGAATGGTAGTGATGATAATCTCAAGTATCTTAATGCTGTTGAGAAATACCTTGATGGTCAGCAAAACTTTGCAATCAGAAGGTATGATAACAACGGTAGAGCTTTATATGATATTAACTTA

>MK673392

ACTTGAAACAATAACAACTTCTCTTCACTGAAGATTACTGGAGAGAACCCAGGATCATTTGGACTAGTAAGAAGCCAAAATGAGAACTTAAACATCGCAAGTGTTACAAAGAATGGTAGTGATGATAATCTCAAGTATCTTAATGCTGTTGAGAAATACCTTGATGGTCAGCAAAACTTTGCAATCAGAAGGTATGATAACAACGGTAGAGCTTTATATGATATTAACTTA

>MK673393

ACTTGAAACAATAACAACTTCTCTTCACTAAAGATTACTGGAGAGAACCCAGGATCATTTGGATTAGTAAGAAGCCAAAATGACAACTTAAATATTTCAAATGTTATTACAAAGAATGATGCTAATGATGATAATCTCAAGTATCTCAATGATGTTGAGAAATACCTTGATGGTCAGCAAAACTTTGCAATCAGAAGGTATGATAACAACGGTAGAGCTTTATATGATATTAACTTA

>MK673394

ACTTGAAACAATAACAACTTCTCTTCACTAAAGATTACTGGAGAGAACCCAGGATCATTTGGATTAGTAAGAAGCCAAAATGACAACTTAAATATTTCAAGTGTTACAAAGAATGTTAATGATGATAATCTCAAGCATCTCAATGCTGTTGAGAAATACCTTGATGGTCAGCAAAACTTTGCAATCAGAAGGTATGATAACAACGGTAGAGCTTTATATGATATTAACTTA

>MK673395

ACTTGAAACAATAACAACTTCTCTTCACTGAAGATTACTGGAGAGAACCCAGGATCATTTGGACTAGTAAGAAGCCAAAATGAGAACTTAAACATCGAAAGTGTTACAAAGAATGGTAGTGATGATAATCTCAAGTATCTTAATGCTGTTGAGAAGTACCTTGATGGTCAGCAAAACTTTGCAATCAGAAGGTATGATAACAACGGTAGAGCTTTATATGATATTAACTTA

>MK673396

ACTTGAAACAATAACAACTTCTCTTCACTAAAGATTACTGGAGAGAACCCAGGATCATTTGGACTAGTAAGAAGCCAAAATGAGAACTTAAACATCGCAAGTGTTACAAAGAATGGTAGTGATGATAATCTCAAGTATCTCAATGCTGTTGAGAAATACCTTGATGGTCAGCAAAACTTTGCAATCAGAAGGTATGATAACAACGGTAGAGCTTTATATGATATTAACTTA

>MK673397

acttgaaacaataacaacttctcttcactaaagattactggagagaacccaggatcatttggattagtaagaagccaaaatgacaacttaaatatttcaaatgttattacaaagaatgatgctaatgatgataatctcaagtatctcaatgatgttgagaaataccttgatggtcagcaaaactttgcaatcagaaggtatgataacaacggtagagctttatatgatattaactta

>MK673398

ACTTGAAACAATAACAACTTCTCTTCACTAAAGATTACTGGAGAGAACCCAGGATCATTTGGATTAGTAAGAAGCCAAAATGACAACTTAAATATTTCAAATGTTATTACAAAGAATGATGCTAATGATGATAATCTCAAGTATCTCAATGATGTTGAGAAATACCTTGATGGTCAGCAAAACTTTGCAATCAGAAGGTATGATAACAACGGTAGAGCTTTATATGATATTAACTTA

>MK673399

ACTTGAAACAATAACAACTTCTCTTCACTAAAGATTACTGGAGAGAACCCAGGATCATTTGGATTAGTAAGAAGCCAAAATGACAACTTAAATATTTCAAATGTTACAAAGAATGTTAGTGATGATAATCTCAAGTATCTCAATTATGTTGAGAAATACCTTGATGGTCAGCAAAACTTTGCAATCAGAAGGTATGATAACAACGGTAGAGCTTTATATGATATTAACTTA

>MK673400

ACTTGAAACAATAACAACTTCTCTTCACTAAAGATTACTGGAGAGAACCCAGGATCATTTGGATTAGTAAGAAGCCAAAATGACAACTTAAATATTTCAAATGTTACAAAGAATGTTAGTGATGATAATCTCAAGTATCTCAATTATGTTGAGAAATACCTTGATGGTCAGCAAAACTTTGCAATCAGAAGGTATGATAACAACGGTAGAGCTTTATATGATATTAACTTA

>MK673401

ACTTGAAACAATAACAACTTCTCTTCACTAAAGATTACTGGAGAGAACCCAGGATCATTTGGACTAGTAAGAAGCCAAAATGAGAACTTAAACATCGCAAGTGTTACAAAGAATGATAGTGATGATAATCTCAAGTATCTTAATTCTGTTGAGAAATACCTTGATGGTCAGCAAAACTTTGCAATCAGAAGGTATGATAACAACGGTAGAGCTTTATATGATATTAACTTA

>MK673402

ACTTGAAACAATAACAACTTCTCTTCACTAAAGATTACTGGAGAGAACCCAGGATCATTTGGACTAGTAAGAAGCCAAAATGAGAACTTAAACATCGCAAGTGTTACAAAGAATGATAGTGATGATAATCTCAAGTATCTTAATTCTGTTGAGAAATACCTTGATGGTCAGCAAAACTTTGCAATCAGAAGGTATGATAACAACGGTAGAGCTTTATATGATATTAACTTA

>MK673403

ACTTGAAACAATAACAACTTCTCTTCACTGAAGATTACTGGAGAGAACCCAGGATCATTTGGACTAGTAAGAAGCCAAAATGAGAACTTAAACATCGCAAGTGTTACAAAGAATGATAATGATGATAATCTCAAGTATCTTAATTCTGTTGAGAAATACCTTGATGGTCAGCAAAACTTTGCAATCAGAAGGTATGATAACAACGGTAGAGCTTTATATGATATTAACTTA

>MK673404

ACTTGAAACAATAACAACTTCTCTTCACTGAAGATTACTGGAGAGAACCCAGGATCATTTGGACTAGTAAGAAGCCAAAATGAGAACTTAAACATCGCAAGTGTTACAAAGAATGATAATGATGATAATCTCAAGTATCTTAATTCTGTTGAGAAATACCTTGATGGTCAGCAAAACTTTGCAATCAGAAGGTATGATAACAACGGTAGAGCTTTATATGATATTAACTTA

>MK673405

ACTTGAAACAATAACAACTTCTCTTCACTGAAGATTACTGGAGAGAACCCAGGATCATTTGGACTAGTAAGAAGCCAAAATGAGAACTTAAACATCGCAAGTGTTACAAAGAATGATAATGATGATAATCTCAAGTATCTTAATTCTGTTGAGAAATACCTTGATGGTCAGCAAAACTTTGCAATCAGAAGGTATGATAACAACGGTAGAGCTTTATATGATATTAACTTA

>MK673406

ACTTGAAACAATAACAACTTCTCTTCACTGAAGATTACTGGAGAGAACCCAGGATCATTTGGACTAGTAAGAAGCCAAAATGAGAACTTAAACATCGCAAGTGTTACAAAGAATGATAATGATGATAATCTCAAGTATCTTAATTCTGTTGAGAAATACCTTGATGGTCAGCAAAACTTTGCAATCAGAAGGTATGATAACAACGGTAGAGCTTTATATGATATTAACTTA

>MK673407

ACTTGAAACAATAACAACTTCTCTTCACTAAAGATTACTGGAGAGAACCCAGGATCATTTGGATTAGTAAGAAGCCAAAATGACAACTTAAATATTTCAAATGTTGCAAATGATGATAATAATCTCAAGTATCTCAATGATGTTGAGAAATACCTTGATGGTCAGCAAAACTTTGCAATCAGAAGGTATGATAACAACGGTAGAGCTTTATATGATATTAACTTA

>MK673408

ACTTGAAACAATAACAACTTCTCTTCACTAAAGATTACTGGAGAGAACCCAGGATCATTTGGATTAGTAAGAAGCCAAAATGACAACTTAAATATTTCAAATGTTACAAAGAATGTTAGTGATGATAATCTCAAGTATCTCAATGATTTTGAGAAATACCTTGATGGTCAGCAAAACTTTGCAATCAGAAGGTATGATAACAACGGTAGAGCTTTATATGATATTAACTTA

>MK673409

ACTTGAAACAATAACAACTTCTCTTCACTGAAGATTACTGGAGAGAACCCAGGATCATTTGGACTAGTAAGAAGCCAAAATGAGAACTTAAACATCGCAAGTGTTACAAAGAATGATAGTGATGATAATCTCAAGTATCTTAATTCTGTTGAGAAATACCTTGATGGTCAGCAAAACTTTGCAATCAGAAGGTATGATAACAACGGTAGAGCTTTATATGATATTAACTTA

>MK673410

ACTTGAAACAATAACAACTTCTCTTCACTGAAGATTACTGGAGAGAACCCAGGATCATTTGGACTAGTAAGAAGCCAAAATGAGAACTTAAACATCGCAAGTGTTACAAAGAATGGTAGTGATGATAATCTCAAGTATCTTAATGCTGTTGAGAAATACCTTGATGGTCAGCAAAACTTTGCAATCAGAAGGTATGATAACAACGGTAGAGCTTTATATGATATTAACTTA

>MK673411

ACTTGAAACAATAACAACTTCTCTTCACTGAAGATTACTGGAGAGAACCCAGGATCATTTGGACTAGTAAGAAGCCAAAATGAGAACTTAAACATCGCAAGTGTTACAAAGAATGGTAGTGATGATAATCTCAAGTATCTTAATGCTGTTGAGAAATACCTTGATGGTCAGCAAAACTTTGCAATCAGAAGGTATGATAACAACGGTAGAGCTTTATATGATATTAACTTA

>MK673412

ACTTGAAACAATAACAACTTCTCTTCACTGAAGATTACTGGAGAGAACCCAGGATCATTTGGACTAGTAAGAAGCCAAAATGAGAACTTAAACATCGCAAGTGTTACAAAGAATGGTAGTGATGATAATCTCAAGTATCTTAATGCTGTTGAGAAATACCTTGATGGTCAGCAAAACTTTGCAATCAGAAGGTATGATAACAACGGTAGAGCTTTATATGATATTAACTTA

>MK673413

ACTTGAAACAATAACAACTTCTCTTCACTGAAGATTACTGGAGAGAACCCAGGATCATTTGGACTAGTAAGAAGCCAAAATGAGAACTTAAACATCGCAAGTGTTACAAAGAATGGTAGTGATGATAATCTCAAGTATCTTAATGCTGTTGAGAAATACCTTGATGGTCAGCAAAACTTTGCAATCAGAAGGTATGATAACAACGGTAGAGCTTTATATGATATTAACTTA

>MK673414

ACTTGAAACAATAACAACTTCTCTTCACTAAAGATTACTGGAGAGAACCCAGGATCATTTGGATTAGTAAGAAGCCGAAATGACAACTTAAATATTTCAAGTGTTACAAAGAATGTTAATAATGATAATCTCAAGTATCTCAGTGTTGTTGAGAAATACCTTGATGGTCAGCAAAACTTTGCAATCAGAAGGTATGATAACAACGGTAGAGCTTTATATGATATTAACTTA

>MK673415

ACTTGAAACAATAACAACTTCTCTTCACTAAAGATTACTGGAGAGAACCCAGGATCATTTGGATTAGTAAGAAGCCGAAATGACAACTTAAATATTTCAAGTGTTACAAAGAATGTTAATAATGATAATCTCAAGTATCTCAGTGTTGTTGAGAAATACCTTGATGGTCAGCAAAACTTTGCAATCAGAAGGTATGATAACAACGGTAGAGCTTTATATGATATTAACTTA

>MK673416

ACTTGAAACAATAACAACTTCTCTTCACTAAAGATTACTGGAGAGAACCCAGGATCATTTGGATTAGTAAGAAGCCAAAATGACAACTTAAATATTTCAAGTGTTACAAAGAATGTTAGTGATGATAATCTCAAGTATCTCAATGCTGTTGAGAAATACCTTGATGATCAGCAAAACTTTGCAATCAGAAGGTATGATAACAACGGTAGAGCTTTATATGATATTAACTTA

>MK673417

ACTTGAAACAATAACAACTTCTCTTCACTAAAGATTACTGGAGAGAACCCAGGATCATTTGGATTAGTAAGAAGCCAAAATGACAACTTAAATATTTCAAGTGTTACAAAGAATGTTAATAATGATAATCTCAAGTATCTCAATGCTGTTGAGAAATACCTTGATGGTCAGCAAAACTTTGCAATCAGAAGGTATGATAACAACGGTAGAGCTTTATATGATATTAACTTA

>MK673418

ACTTGAAACAATAACAACTTCTCTTCACTAAAGATTACTGGAGAGAACCCAGGATCATTTGGATTAGTAAGAAGCCAAAATGACAACTTAAATATTTCAAGTGTTACAAAGAATGTTAATGATGATAATCTCAAGTATCTCAATGCTGTTGAGAAATACCTTGATGGTCAGCAAAACTTTGCAATCAGAAGGTATGATAACAACGGTAGAGCTTTATATGATATTAACTTA

>MK673419

ACTTGAAACAATAACAACTTCTCTTCACTAAAGATTACTGGAGAGAACCCAGGATCATTTGGATTAGTAAGAAGCCAAAATGACAACTTAAATATTTCAAGTGTTACAAAGAATGATAGTGATGATAATCTCAAGTATCTCAATGCTGTTGAGAAATACCTTGATGGTCAGCAAAACTTTGCAATCAGAAGGTATGATAACAACGGTAGAGCTTTATATGATATTAACTTA

>MK673420

ACTTGAAACAATAACAACTTCTCTTCACTAAAGATTACTGGAGAGAACCCAGGATCATTTGGATTAGTAAGAAGCCAAAATGACAACTTAAATATTTCAAGTGTTACAAAGAATGTTAGTGATGATAATCTCAAGTATCTCAATGCTGTTGAGAAATACCTTGATGGTCAGCAAAACTTTGCAATCAGAAGGTATGATAACAACGGTAGAGCTTTATATGATATTAACTTA

>MK673421

ACTTGAAACAATAACAACTTCTCTTCACTAAAGATTACTGGAGAGAACCCAGGATCATTTGGATTAGTAAGAAGCCAAAATGACAACTTAAATATTTCAAGTGTTACAAAGAATGTTAGTGATGATAATCTCAAGTATCTCAATGCTGTTGAGAAATACCTTGATGGTCAGCAAAACTTTGCAATCAGAAGGTATGATAACAACGGTAGAGCTTTATATGATATTAACTTA

>MK673422

ACTTGAAACAATAACAACTTCTCTTCACTAAAGATTACTGGAGAGAACCCAGGATCATTTGGACTAGTAAGAAGCCAAAATGAGAACTTAAACATCGCAAGTGTTACAAAGAATGGTAGTGATGATAATCTCAAGTATCTCAATGCTGTTGAGAAATACCTTGATGGTCAGCAAAACTTTGCAATCAGAAGGTATGATAACAACGGTAGAGCTTTATATGATATTAACTTA

>MK673423

ACTTGAAACAATAACAACTTCTCTTCACTGAAGATTACTGGAGAGAACCCAGGATCATTTGGACTAGTAAGAAGCCAAAATGAGAACTTAAACATCGCAAGTGTTACAAAGAATGATAGTGAGGATAATCTCAAGTATCTTAATTCTGTTGAGAAATACCTTGATGGTCAGCAAAACTTTGCAATCAGAAGGTATGATAACAACGGTAGAGCTTTATATGATATTAACTTA

>MK673424

ACTTGAAACAATAACAACTTCTCTTCACTGAAGATTACTGGAGAGAACCCAGGATCATTTGGACTAGTAAGAAGCCAAAATGAGAACTTAAACATCGCAAGTGTTACAAAGAATGATAGTGATGATAATCTCAAGTATCTTAATTCTGTTGAGAAATACCTTGATGGTCAGCAAAACTTTGCAATCAGAAGGTATGATAACAACGGTAGAGCTTTATATGATATTAACTTA

>MK673425

ACTTGAAACAATAACAACTTCTCTTCACTAAAGATTACTGGAGAGAACCCAGGATCATTTGGATTAGTAAGAAGCCAAAATGACAACTTAAACATCGCAAGTGTTACAAAGAATGTTAGTGATGATAATCTCAAGTATCTCAATGCTGTTGAGAAATACCTTGATGGTCAGCAAAACTTTGCAATCAGAAGGTATGATAACAACGGTAGAGCTTTATATGATATTAACTTA

>MK673426

ACTTGAAACAATAACAACTTCTCTTCACTAAAGATTACTGGAGAGAACCCAGGATCATTTGGATTAGTAAGAAGCCAAAATGACAACTTAAATATTTCAAGTGTTACAAAGAATGTTAGTGATGATAATCTCAAGTATCTTAATAATGTTGAGAAATACCTTGATGGTCAGCAAAACTTTGCAATCAGAAGGTATGATAACAACGGTAGAGCTTTATATGATATTAACTTA

>MK673427

ACTTGAAACAATAACAACTTCTCTTCACTAAAGATTACTGGAGAGAACCCAGGATCATTTGGATTAGTAAGAAGCCAAAATGACAACTTAAATATTTCAAATGTTACAAAGAATGTTAGTGATGATAATCTCAAGTATCTCAATTATGTTGAGAAATACCTTGATGATCAGCAAAACTTTGCAATCAGAAGGTATGATAACAACGGTAGAGCTTTATATGATATTAACTTA

>MK673428

ACTTGAAACAATAACAACTTCTCTTCACTAAAGATTACTGGAGAGAACCCAGGATCATTTGGATTAGTAAGAAGCCAAAATGACAACTTAAATATTTCAAGTGTTACAAAGAATGTTAGTGATGATAATCTCAAGTATCTCAATGCTGTTGAGAAATACCTTGATGGTCAGCAAAACTTTGCAATCAGAAGGTATGATAACAACGGTAGAGCTTTATATGATATTAACTTA

>MK673429

ACTTGAAACAATAACAACTTCTCTTCACTGAAGATTACTGGAGAGAACCCAGGATCATTTGGATTAGTAAGAAGCCAAAATGCGAACTTAAATATCGCAAGTGTTCAAAAGCAATCAGGTGATGATAATCTCAAGTATCTTAATGCTGTTGAGAAATACCTTGATGGTCAGCAAAACTTTGCAATCAGAAGGTATGATAACAACGGTAGAGCTTTATATGATATTAACTTA

>MK673430

ACTTGAAACAATAACAACTTCTCTTCACTAAAGATTACTGGAGAGAACCCAGGATCATTTGGATTAGTAAGAAGCCAAAATGACAACTTAAATATTTCAAGTGTTACAAAGAATGTTGGTGATGATAATCTCAAGTATCTCAATGCTGTTGAGAAATACCTTGATGGTCAGCAAAACTTTGCAATCAGAAGGTATGATAACAACGGTAGAGCTTTATATGATATTAACTTA

>MK673431

ACTTGAAACAATAACAACTTCTCTTCACTGAAGATTACTGGAGAGAACCCAGGATCATTTGGATTAGTAAGAAGCCAAAATGCGAACTTAAATATCGCAAGTGTTCAAAAGCAATCAGGTGATGATAATCTCAAGTATCTTAATGCTGTTGAGAAATACCTTGATGGTCAGCAAAACTTTGCAATCAGAAGGTATGATAACAACGGTAGAGCTTTATATGATATTAACTTA

>MK673432

ACTTGAAACAATAACAACTTCTCTTCACTAAAGATTACTGGAGAGAACCCAGGATCATTTGGACTAGTAAGAAGCCAAAATGAGAACTTAAACATCGCAAGTGTTACAAAGAATGGTAGTGATGATAATCTCAAGTATCTCAATGCTGTTGAGAAATACCTTGATGGTCAGCAAAACTTTGCAATCAGAAGGTATGATAACAACGGTAGAGCTTTATATGATATTAACTTA

>MK673433

ACTTGAAACAATAACAACTTCTCTTCACTGAAGATTACTGGAGAGAACCCAGGATCATTTGGACTAGTAAGAAGCCAAAATGAGAACTTAAACATCGCAAGTGTTACAAAGAATGGTAGTGATGATAATCTCAAGTATCTTAATGCTGTTGAGAAATACCTTGATGGTAAGCAAAACTTTGCAATCAGAAGGTATGATAACAACGGTAGAGCTTTATATGATATTAACTTA

>MK673434

ACTTGAAACAATAACAACTTCTCTTCACTAAAGATTACTGGAGAGAACCCAGGATCATTTGGACTAGTAAGAAGCCAAAATGAGAACTTAAACATCGCAAGTGTTACAAAGAATGGTAGTGATGATAATCTCAAGTATCTCAATGCTGTTGAGAAATACCTTGATGGTCAGCAAAACTTTGCAATCAGAAGGTATGATAACAACGGTAGAGCTTTATATGATATTAACTTA

>MK673435

ACTTGAAACAATAACAACTTCTCTTCACTAAAGATTACTGGAGAGAACCCAGGATCATTTGGACTAGTAAGAAGCCAAAATGAGAACTTAAACATCGCAAGTGTTACAAAGAATGGTAGTGATGATAATCTCAAGTATCTCAATGCTGTTGAGAAATACCTTGATGGTCAGCAAAACTTTGCAATCAGAAGGTATGATAACAACGGTAGAGCTTTATATGATATTAACTTA

>MK673436

ACTTGAAACAATAACAACTTCTCTTCACTAAAGATTACTGGAGAGAACCCAGGATCATTTGGATTAGTAAGAAGCCAAAATGACAACTTAAATATTTCAAATGTTATTACAAAGAATAATGTTAGTGATGATAATCTCAAGTATCTCAATGATGTTGAGAAATACCTTGATGATCGGCAAAACTTTGCAATCAGAAGGTATGATAACAACGGTAGAGCTTTATATGATATTAACTTA

>MK673437

ACTTGAAACAATAACAACTTCTCTTCACTAAAGATTACTGGAGAGAACCCAGGATCATTTGGACTAGTAAGAAGCCAAAATGAGAACTTAAACATCGCAAGTGTTACAAAGAATGGTAGTGATGATAATCTCAAGTATCTCAATGCTGTTGAGAAATACCTTGATGGTCAGCAAAACTTTGCAATCAGAAGGTATGATAACAACGGTAGAGCTTTATATGATATTAACTTA

>MK673438

ACTTGAAACAATAACAACTTCTCTTCACTAAAGATTACTGGAGAGAACCCAGGATCATTTGGACTAGTAAGAAGCCAAAATGAGAACTTAAACATCGCAACTGTTACAAAGAATAATAGTGATGATAATCTCAAGTATCTTAATTCTGTTGAGAAATACCTTGATGGTCAGCAAAACTTTGCAATCAGAAGGTATGATAACAACGGTAGAGCTTTATATGATATTAACTTA

>MK673439

ACTTGAAACAATAACAACTTCTCTTCACTAAAGATTACTGGAGAGAACCCAGGATCATTTGGATTAGTAAGAAGCCAAAATGACAACTTAAATATTTCAAGTGTTACAAAGAATGTTAGTGATGATAATCTCAAGTATCTCAATGCTGTTGAGAAATACCTTGATGGTCAACAAAACTTTGCAATCAGAAGGTATGATAACAACGGTAGAGCTTTATATGATATTAACTTA

>MK673440

ACTTGAAACAATAACAACTTCTCTTCACTAAAGATTACTGGAGAGAACCCAGGATCATTTGGATTAGTAAGAAGCCAAAATGACAACTTAGACATCGCAAGTGTTACAAAGGATGCTAGTGATGATAATCTCAAGTATCTCAATGCTGTTGAGAAATACCTTGATGGTCAGCAAAACTTTGCAATCAGAAGGTATGATAACAACGGTAGAGCTTTATATGATATTAACTTA

>MK673441

ACTTGAAACAATAACAACTTCTCTTCACTAAAGATTACTGGAGAGAACCCAGGATCATTTGGACTAGTAAGAAGCCAAAATGAGAACTTAAACATCGCAAGTGTTACAAAGAATGATAGTGATGATAATCTCAAGTATCTTAATTCTGTTGAGAAATACCTTGATGGTCAGCAAAACTTTGCAATCAGAAGGTATGATAACAACGGTAGAGCTTTATATGATATTAACTTA

>MK673442

ACTTGAAACAATAACAACTTCTCTTCACTGAAGATTACTGGAGAGAACCCAGGATCATTTGGACTAGTAAGAAGCCAAAATGAGAACTTAAACATCGCAAGTGTTACAAAGAATGGTAGTGATGATAATCTCAAGTATCTTAATGCTGTTGAGAAATACCTTGATGGTCAGCAAAACTTTGCAATCAGAAGGTATGATAACAACGGTAGAGCTTTATATGATATTAACTTA

>MK673443

ACTTGAAACAATAACAACTTCTCTTCACTAAAGATTACTGGAGAGAACCCAGGATCATTTGGATTAGTAAGAAGCCAAAATGACAACTTAAATATTTCAAATGTTACAAAGAATGTTAGTGATGATAATCTCAAGTATCTCAATGATGTTGAGAAATACCTTGATGGTCAGCAAAACTTTGCAATCAGAAGGTATGATAACAACGGTAGAGCTTTATATGATATTAACTTA

>MK673444

ACTTGAAACAATAACAACTTCTCTTCACTAAAGATTACTGGAGAGAACCCAGGATCATTTGGATTAGTAAGAAGCCAAAATGACAACTTAAATATTTCAAGTGTTACAAAGAATGTTAGTGATGATAATCTCAAGTATCTCAATGCTGTTGAGAAATACCTTGATGGTCAGCAAAACTTTGCAATCAGAAGGTATGATAACAACGGTAGAGCTTTATATGATATTAACTTA

>MK673445

ACTTGAAACAATAACAACTTCTCTTCACTAAAGATTACTGGAGAGAACCCAGGATCATTTGGATTAGTAAGAAGCCAAAATGACAACTTAAATATTTCAAATGTTATTACAAAGAATAATGTTAGTGATGATAATCTCAAGTATCTCAATGATGTTGAGAAATACCTTGATGGTCAGCAAAACTTTGCAATCAGAAGGTATGATAACAACGGTAGAGCTTTATATGATATTAACTTA

>MK673446

ACTTGAAACAATAACAACTTCTCTTCACTGAAGATTACTGGAGAGAACCCAGGATCATTTGGACTAGTAAGAAGCCAAAATGAGAACTTAAACATCGCAAGTGTTACAAAGAATGGTAGTGATGATAATCTCAAGTATCTTAATGCTGTTGAGAAGTACCTTGATGGTCAGCAAAACTTTGCAATCAGAAGGTATGATAACAACGGTAGAGCTTTATATGATATTAACTTA

MultAlin

Name: 1.G37(L43967)[1]	Len: 246 Check: 2758 Weight: 0.99

Name: 1.MgPa[1]	Len: 246 Check: 2758 Weight: 0.99

Name: 53.H-P9300A191[53] Len: 246 Check: 2430 Weight: 1.01

Name: 95.KC445152[95]	Len: 246 Check: 2550 Weight: 1.01

Name: 32.M2288[32]	Len: 246 Check: 2570 Weight: 1.06

Name: 2.M2341[2]	Len: 246 Check: 2839 Weight: 0.96

Name: 0.DQ248087[2]	Len: 246 Check: 2839 Weight: 0.96

Name: 0.DQ248095[2]	Len: 246 Check: 2839 Weight: 0.96

Name: 0.KC445148[2]	Len: 246 Check: 2839 Weight: 0.96

Name: MK673342	Len: 246 Check: 2839 Weight: 0.96

Name: MK673343	Len: 246 Check: 2839 Weight: 0.96

Name: MK673359	Len: 246 Check: 2839 Weight: 0.96

Name: MK673360	Len: 246 Check: 2839 Weight: 0.96

Name: MK673363	Len: 246 Check: 2839 Weight: 0.96

Name: MK673364	Len: 246 Check: 2839 Weight: 0.96

Name: MK673389	Len: 246 Check: 2839 Weight: 0.96

Name: MK673420	Len: 246 Check: 2839 Weight: 0.96

Name: MK673421	Len: 246 Check: 2839 Weight: 0.96

Name: MK673428	Len: 246 Check: 2839 Weight: 0.96

Name: MK673444	Len: 246 Check: 2839 Weight: 0.96

Name: 74.FJ750844*	Len: 246 Check: 2839 Weight: 0.96

Name: 22.314F_RUS[22]	Len: 246 Check: 2953 Weight: 0.96

Name: MK673344	Len: 246 Check: 2953 Weight: 0.96

Name: MK673345	Len: 246 Check: 2953 Weight: 0.96

Name: MK673430	Len: 246 Check: 2953 Weight: 0.96

Name: 23.45/01-UK_PR	Len: 246 Check: 2905 Weight: 0.98

Name: 0.KC445158[23]	Len: 246 Check: 2905 Weight: 0.98

Name: 66.FJ750834	Len: 246 Check: 2899 Weight: 0.99

Name: 82.HM565866[82]	Len: 246 Check: 3133 Weight: 0.99

Name: MK673419	Len: 246 Check: 2516 Weight: 1.01

Name: 19.LF-P7262,P1[19] Len: 246 Check: 2575 Weight: 1.01

Name: 21.2094_UME[21]	Len: 246 Check: 2563 Weight: 0.99

Name: 0.KC445144[21]	Len: 246 Check: 2563 Weight: 0.99

Name: MK673379	Len: 246 Check: 2749 Weight: 0.99

Name: MK673439	Len: 246 Check: 2749 Weight: 0.99

Name: MK673418	Len: 246 Check: 2719 Weight: 1.01

Name: 10.H7755G1[10]	Len: 246 Check: 2857 Weight: 1.01

Name: 25.RYH217[25]	Len: 246 Check: 2773 Weight: 0.99

Name: MK673416	Len: 246 Check: 2773 Weight: 0.99

Name: 20.M2300[20]	Len: 246 Check: 3125 Weight: 1.04

Name: 86.HM565870[86]	Len: 246 Check: 2680 Weight: 1.04

Name: 5.LF-P6257,J11[5]	Len: 246 Check: 2745 Weight: 1.01

Name: 0.FJ750839[5]	Len: 246 Check: 2745 Weight: 1.01

Name: 9b.M6285_R65[9]	Len: 246 Check: 2901 Weight: 1.02

Name: 16.LF-P6601,M1[16] Len: 246 Check: 2859 Weight: 1.04

Name: 81.HM565865[81]	Len: 246 Check: 2924 Weight: 1.06

Name: 0.KC445142[90b]	Len: 246 Check: 3145 Weight: 1.02

Name: 0.KC445150[90b]	Len: 246 Check: 3145 Weight: 1.02

Name: 17.RYH214[17]	Len: 246 Check: 1946 Weight: 1.06

Name: 28.M6270_D24-A[28] Len: 246 Check: 1920 Weight: 1.06

Name: 83.HM565867[83]	Len: 246 Check: 2443 Weight: 1.06

Name: MK673394	Len: 246 Check: 2090 Weight: 1.06

Name: MK673417	Len: 246 Check: 2587 Weight: 1.06

Name: 27.35M_RUS[27]	Len: 246 Check: 2545 Weight: 1.07

Name: 8.66-0[8]	Len: 246 Check: 2481 Weight: 1.02

Name: 0.KC445146[8]	Len: 246 Check: 2481 Weight: 1.02

Name: MK673443	Len: 246 Check: 2481 Weight: 1.02

Name: 24.5336_D[24]	Len: 246 Check: 2349 Weight: 1.04

Name: 0.DQ248088[24]	Len: 246 Check: 2349 Weight: 1.04

Name: 0.KC445156[24]	Len: 246 Check: 2349 Weight: 1.04

Name: 15.DK523[15]	Len: 246 Check: 3031 Weight: 1.09

Name: MK673408	Len: 246 Check: 3118 Weight: 1.11

Name: 93.KC445147[93]	Len: 246 Check: 2739 Weight: 1.11

Name: 29.1-0[29]	Len: 246 Check: 2445 Weight: 1.11

Name: 88.HM565872[88]	Len: 246 Check: 3272 Weight: 1.12

Name: 26.RYH117[26]	Len: 246 Check: 3744 Weight: 1.14

Name: 18.76-0[18]	Len: 246 Check: 3343 Weight: 1.09

Name: 79.DQ248093	Len: 246 Check: 2469 Weight: 1.09

Name: MK673399	Len: 246 Check: 3079 Weight: 1.04

Name: MK673400	Len: 246 Check: 3079 Weight: 1.04

Name: 70.FJ750840	Len: 246 Check: 3013 Weight: 1.09

Name: MK673427	Len: 246 Check: 3013 Weight: 1.09

Name: 6.DK_PR9[6]	Len: 246 Check: 1044 Weight: 0.89

Name: 0.FJ750838[6]	Len: 246 Check: 1044 Weight: 0.89

Name: MK673348	Len: 246 Check: 1044 Weight: 0.89

Name: MK673349	Len: 246 Check: 1044 Weight: 0.89

Name: MK673361	Len: 246 Check: 1044 Weight: 0.89

Name: MK673362	Len: 246 Check: 1044 Weight: 0.89

Name: MK673383	Len: 246 Check: 1044 Weight: 0.89

Name: MK673384	Len: 246 Check: 1044 Weight: 0.89

Name: MK673425	Len: 246 Check: 1044 Weight: 0.89

Name: 76.DQ248090*	Len: 246 Check: 1044 Weight: 0.89

Name: 30.H346J1[30]	Len: 246 Check: 1158 Weight: 0.93

Name: 90.JQ439992[90]	Len: 246 Check: 1104 Weight: 0.93

Name: MK673357	Len: 246 Check: 840 Weight: 0.89

Name: MK673358	Len: 246 Check: 840 Weight: 0.89

Name: 11.M2321[11]	Len: 246 Check: 823 Weight: 0.91

Name: MK673365	Len: 246 Check: 823 Weight: 0.91

Name: MK673366	Len: 246 Check: 823 Weight: 0.91

Name: MK673385	Len: 246 Check: 459 Weight: 0.93

Name: MK673386	Len: 246 Check: 459 Weight: 0.93

Name: 65.FJ750832	Len: 246 Check: 755 Weight: 0.96

Name: MK673440	Len: 246 Check: 1019 Weight: 0.96

Name: 31.DK15776[31]	Len: 246 Check: 979 Weight: 1.04

Name: 77.DQ248091	Len: 246 Check: 9849 Weight: 1.06

Name: 12.H9504K1[12]	Len: 246 Check: 3483 Weight: 1.17

Name: 61.FJ750827	Len: 246 Check: 4151 Weight: 1.15

Name: MK673426	Len: 246 Check: 3183 Weight: 1.15

Name: 0.KC445143[91b]	Len: 246 Check: 2009 Weight: 1.22

Name: MK673377	Len: 246 Check: 2205 Weight: 1.12

Name: MK673378	Len: 246 Check: 2205 Weight: 1.12

Name: MK673414	Len: 246 Check: 3770 Weight: 1.12

Name: MK673415	Len: 246 Check: 3770 Weight: 1.12

Name: 36.1182_UME[36]	Len: 246 Check: 2623 Weight: 1.30

Name: 37.H12202O1[37]	Len: 246 Check: 1406 Weight: 1.30

Name: 71.FJ750841	Len: 246 Check: 3314 Weight: 1.32

Name: 35.RYH70[35]	Len: 246 Check: 2375 Weight: 1.32

Name: 89.JQ439991[89a]	Len: 246 Check: 991 Weight: 1.38

Name: 3.H-P7791B11[3]	Len: 246 Check: 2048 Weight: 0.88

Name: 0.KC445139[3]	Len: 246 Check: 2048 Weight: 0.88

Name: 0.KC445141[3]	Len: 246 Check: 2048 Weight: 0.88

Name: 0.KC445160[3]	Len: 246 Check: 2048 Weight: 0.88

Name: MK673446	Len: 246 Check: 2048 Weight: 0.88

Name: 78.DQ248092*	Len: 246 Check: 2048 Weight: 0.88

Name: 46.RYH181[46]	Len: 246 Check: 2312 Weight: 0.91

Name: 42.H12755V1[42]	Len: 246 Check: 1964 Weight: 0.93

Name: 45.399F_RUS[45]	Len: 246 Check: 1958 Weight: 0.94

Name: 47.18-0R[47]	Len: 246 Check: 1928 Weight: 0.94

Name: 60.EU131382	Len: 246 Check: 1946 Weight: 0.94

Name: MK673371	Len: 246 Check: 1706 Weight: 0.89

Name: MK673372	Len: 246 Check: 1706 Weight: 0.89

Name: MK673380	Len: 246 Check: 1706 Weight: 0.89

Name: MK673390	Len: 246 Check: 1706 Weight: 0.89

Name: MK673391	Len: 246 Check: 1706 Weight: 0.89

Name: MK673392	Len: 246 Check: 1706 Weight: 0.89

Name: MK673410	Len: 246 Check: 1706 Weight: 0.89

Name: MK673411	Len: 246 Check: 1706 Weight: 0.89

Name: MK673412	Len: 246 Check: 1706 Weight: 0.89

Name: MK673413	Len: 246 Check: 1706 Weight: 0.89

Name: MK673442	Len: 246 Check: 1706 Weight: 0.89

Name: 75.DQ248089	Len: 246 Check: 1972 Weight: 0.93

Name: 80.DQ248094	Len: 246 Check: 2068 Weight: 0.93

Name: 92.KC445145[92]	Len: 246 Check: 2269 Weight: 0.96

Name: 43.123-0[43]	Len: 246 Check: 2646 Weight: 0.96

Name: 13.LF-P7523,O1[13] Len: 246 Check: 2022 Weight: 0.98

Name: MK673433	Len: 246 Check: 1680 Weight: 0.98

Name: 44.168-0[44]	Len: 246 Check: 1954 Weight: 0.94

Name: 0.KC445157[44]	Len: 246 Check: 1954 Weight: 0.94

Name: MK673395	Len: 246 Check: 1966 Weight: 0.98

Name: 7.M6271_E17-AU[7]	Len: 246 Check: 812 Weight: 0.88

Name: 0.FJ750830[7]	Len: 246 Check: 812 Weight: 0.88

Name: MK673346	Len: 246 Check: 812 Weight: 0.88

Name: MK673347	Len: 246 Check: 812 Weight: 0.88

Name: MK673354	Len: 246 Check: 812 Weight: 0.88

Name: MK673355	Len: 246 Check: 812 Weight: 0.88

Name: MK673356	Len: 246 Check: 812 Weight: 0.88

Name: MK673369	Len: 246 Check: 812 Weight: 0.88

Name: MK673370	Len: 246 Check: 812 Weight: 0.88

Name: MK673387	Len: 246 Check: 812 Weight: 0.88

Name: MK673396	Len: 246 Check: 812 Weight: 0.88

Name: MK673422	Len: 246 Check: 812 Weight: 0.88

Name: MK673432	Len: 246 Check: 812 Weight: 0.88

Name: MK673434	Len: 246 Check: 812 Weight: 0.88

Name: MK673435	Len: 246 Check: 812 Weight: 0.88

Name: MK673437	Len: 246 Check: 812 Weight: 0.88

Name: 63.FJ750829	Len: 246 Check: 992 Weight: 0.91

Name: 94.KC445151[94]	Len: 246 Check: 746 Weight: 0.93

Name: 97.KC445155[97]	Len: 246 Check: 903 Weight: 0.94

Name: 14.M6286_R67[14]	Len: 246 Check: 890 Weight: 0.96

Name: 0.KC445140[89b]	Len: 246 Check: 1322 Weight: 0.96

Name: 49.DK11844[49]	Len: 246 Check: 2029 Weight: 1.06

Name: 4.LF-P6486,L11[4]	Len: 246 Check: 2202 Weight: 0.91

Name: 0.FJ750833[4]	Len: 246 Check: 2202 Weight: 0.91

Name: 0.KC445149[4]	Len: 246 Check: 2202 Weight: 0.91

Name: 0.KC445153[4]	Len: 246 Check: 2202 Weight: 0.91

Name: MK673350	Len: 246 Check: 2202 Weight: 0.91

Name: MK673351	Len: 246 Check: 2202 Weight: 0.91

Name: MK673367	Len: 246 Check: 2202 Weight: 0.91

Name: MK673368	Len: 246 Check: 2202 Weight: 0.91

Name: MK673373	Len: 246 Check: 2202 Weight: 0.91

Name: MK673374	Len: 246 Check: 2202 Weight: 0.91

Name: MK673381	Len: 246 Check: 2202 Weight: 0.91

Name: MK673382	Len: 246 Check: 2202 Weight: 0.91

Name: MK673409	Len: 246 Check: 2202 Weight: 0.91

Name: MK673424	Len: 246 Check: 2202 Weight: 0.91

Name: 85.HM565869[85]	Len: 246 Check: 2639 Weight: 0.94

Name: 38.N4219[38]	Len: 246 Check: 2082 Weight: 0.93

Name: MK673403	Len: 246 Check: 2082 Weight: 0.93

Name: MK673404	Len: 246 Check: 2082 Weight: 0.93

Name: MK673405	Len: 246 Check: 2082 Weight: 0.93

Name: MK673406	Len: 246 Check: 2082 Weight: 0.93

Name: 39.129-0[39]	Len: 246 Check: 2136 Weight: 0.96

Name: 62.FJ750828	Len: 246 Check: 2022 Weight: 0.93

Name: MK673401	Len: 246 Check: 2022 Weight: 0.93

Name: MK673402	Len: 246 Check: 2022 Weight: 0.93

Name: MK673441	Len: 246 Check: 2022 Weight: 0.93

Name: MK673423	Len: 246 Check: 1890 Weight: 0.98

Name: 91.JQ439993[91a]	Len: 246 Check: 2196 Weight: 0.99

Name: 64.FJ750831	Len: 246 Check: 2174 Weight: 1.02

Name: 41.RYH115[41]	Len: 246 Check: 2178 Weight: 1.04

Name: 48.TAQMAN3672[48]	Len: 246 Check: 1914 Weight: 1.09

Name: 57.EU131379	Len: 246 Check: 1770 Weight: 1.09

Name: MK673438	Len: 246 Check: 1750 Weight: 1.15

Name: 55.H8692[55]	Len: 246 Check: 2510 Weight: 1.15

Name: 40.RYH23[40]	Len: 246 Check: 1814 Weight: 1.14

Name: 54.H97050G2[54]	Len: 246 Check: 2761 Weight: 1.14

Name: 96.KC445154[96]	Len: 246 Check: 2030 Weight: 1.22

Name: 50.LF-P7551,S1[50] Len: 246 Check: 1903 Weight: 1.24

Name: 87.HM565871[87]	Len: 246 Check: 3399 Weight: 1.35

Name: 98.KC445159[98]	Len: 246 Check: 1107 Weight: 1.37

Name: 73.FJ750843	Len: 246 Check: 3474 Weight: 1.53

Name: 51.111-0[51]	Len: 246 Check: 2224 Weight: 0.96

Name: MK673429	Len: 246 Check: 2224 Weight: 0.96

Name: MK673431	Len: 246 Check: 2224 Weight: 0.96

Name: 59.EU131381	Len: 246 Check: 2044 Weight: 1.01

Name: 67.FJ750835	Len: 246 Check: 3040 Weight: 1.02

Name: 52.H13051F1[52]	Len: 246 Check: 2780 Weight: 1.04

Name: 84.HM565868[84]	Len: 246 Check: 2558 Weight: 1.04

Name: 58.EU131380	Len: 246 Check: 2428 Weight: 1.07

Name: 72.FJ750842	Len: 246 Check: 3229 Weight: 1.11

Name: 9a.DK16221[9]	Len: 246 Check: 3647 Weight: 1.32

Name: 34.19-0[34]	Len: 246 Check: 4777 Weight: 1.27

Name: MK673352	Len: 246 Check: 3716 Weight: 0.75

Name: MK673353	Len: 246 Check: 3716 Weight: 0.75

Name: MK673388	Len: 246 Check: 3716 Weight: 0.75

Name: MK673445	Len: 246 Check: 3716 Weight: 0.75

Name: MK673436	Len: 246 Check: 3734 Weight: 0.81

Name: MK673375	Len: 246 Check: 3385 Weight: 0.73

Name: MK673376	Len: 246 Check: 3385 Weight: 0.73

Name: MK673393	Len: 246 Check: 3385 Weight: 0.73

Name: MK673397	Len: 246 Check: 3385 Weight: 0.73

Name: MK673398	Len: 246 Check: 3385 Weight: 0.73

Name: 68.FJ750836	Len: 246 Check: 1498 Weight: 1.81

Name: 69.FJ750837	Len: 246 Check: 8388 Weight: 1.97

Name: MK673407	Len: 246 Check: 379 Weight: 1.97

Name: 33.M6328_R66[33]	Len: 246 Check: 6353 Weight: 2.24
