# Supplementary material for: Molecular Typing of Mycoplasma genitalium-Positive Specimens Discriminates between Persistent and Recurrent Infections in Cases of Treatment Failure and Supports Contact Tracing
Source: Microorganisms. 2019 Nov 23;7(12):609. doi: 10.3390/microorganisms7120609 (PMC6955785; doi:10.3390/microorganisms7120609)
Supplement: Supplementary file 1 [file microorganisms-07-00609-s001.zip › microorganisms-621513-supple.zip!/3.FigS1.docx]

# Figure S1. Phylogenetic analysis of the adhesin *mgpB* gene (MG191 locus, 281 bp) sequences of 105 *Mycoplasma genitalium*-positive urogenital samples collected in Gipuzkoa (Spain).

# The sequences were identified with the sample number (GenBank accession strain no. in parentheses), the group number of the study (G1-4), the number of the case (C1-31) and sample (S1-4) within the group (date of detection in parentheses). G1: first sample sensitive and second resistant to macrolides. G2: first and second samples both sensitive or both resistant. G3: couples/groups of sexual contacts. G4: not related and randomly distributed samples. Some sequences were from samples that could be in G1 and G3 (Table 2), and G4 is represented in blue. The tree was constructed in MEGA X using the neighbour-joining method with 1000 bootstrap replications for branch support and shows bootstrap values higher than 70 in the branches. The distance is expressed as the number of the nucleotide substitutions per site. To the right of the phylogenetic tree, a column has been included listing the genotypes previously assigned (1-98) in GenBank (references 4, 9-14) and those newly detected in this study (99-120) as a function of their genetic identity (Table S1).

* *M. genitalium* G37 (GenBank accession no. L43967, genotype 1) was used as a reference strain to root the tree.
