# Supplementary material for: Molecular Typing of Mycoplasma genitalium-Positive Specimens Discriminates between Persistent and Recurrent Infections in Cases of Treatment Failure and Supports Contact Tracing
Source: Microorganisms. 2019 Nov 23;7(12):609. doi: 10.3390/microorganisms7120609 (PMC6955785; doi:10.3390/microorganisms7120609)
Supplement: Supplementary file 1 [file microorganisms-07-00609-s001.zip › microorganisms-621513-supple.zip!/4.FigS2.docx]

**Figure S2.** Variable number tandem repeat analysis of lipoprotein gene (MG309 locus) sequences of 74 *Mycoplasma genitalium*-positive urogenital samples collected in Gipuzkoa (Spain).

The nucleotide alignment includes a small portion of the amplified region (~350 bp), corresponding in the *Mycoplasma genitalium* reference strain G37 (GenBank accession no. L43967*) to nucleotides 1714-1767 (the first and last 9 nucleotides, in yellow). The boxed regions represent the trinucleotide repeats (ACT, in green; ATT, in blue), the variations in which were used to identify the 21 types finally detected. Crosses (in grey) indicate gaps introduced to optimise alignment. Schematic representation based on reference 8 from Ma et al.
